# Augmented Memory: Sample-Efficient Generative Molecular Design with Reinforcement Learning

**DOI:** 10.1021/jacsau.4c00066

**Published:** 2024-04-10

**Authors:** Jeff Guo, Philippe Schwaller

**Affiliations:** † Laboratory of Artificial Chemical Intelligence (LIAC), Institut des Sciences et Ingénierie Chimiques, Ecole Polytechnique Fédérale de Lausanne (EPFL), Lausanne 1015, Switzerland; ‡ National Centre of Competence in Research (NCCR) Catalysis, Ecole Polytechnique Fédérale de Lausanne (EPFL), Lausanne 1015, Switzerland

**Keywords:** generative molecular design, sample efficiency, drug discovery, materials design, reinforcement learning

## Abstract

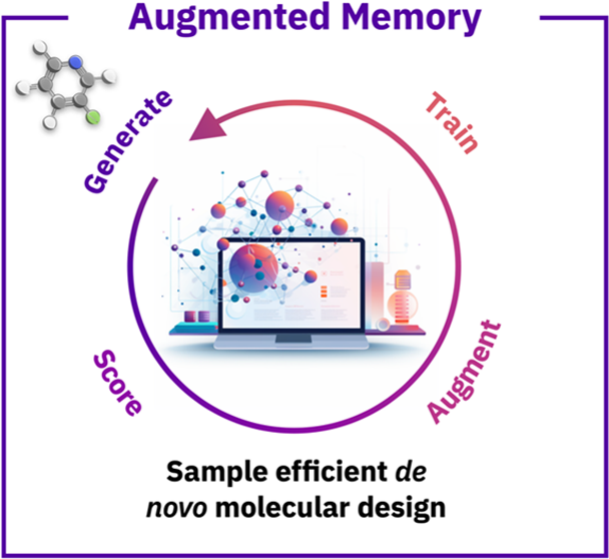

Sample efficiency is a fundamental challenge in *de novo* molecular design. Ideally, molecular generative models should learn to satisfy a desired objective under minimal calls to oracles (computational property predictors). This problem becomes more apparent when using oracles that can provide increased predictive accuracy but impose significant computational cost. Consequently, designing molecules that are optimized for such oracles cannot be achieved under a practical computational budget. Molecular generative models based on simplified molecular-input line-entry system (SMILES) have shown remarkable sample efficiency when coupled with reinforcement learning, as demonstrated in the practical molecular optimization (PMO) benchmark. Here, we first show that experience replay drastically improves the performance of multiple previously proposed algorithms. Next, we propose a novel algorithm called Augmented Memory that combines data augmentation with experience replay. We show that scores obtained from oracle calls can be reused to update the model multiple times. We compare Augmented Memory to previously proposed algorithms and show significantly enhanced sample efficiency in an exploitation task, a drug discovery case study requiring both exploration and exploitation, and a materials design case study optimizing explicitly for quantum-mechanical properties. Our method achieves a new state-of-the-art in sample-efficient *de novo* molecular design, outperforming all of the previously reported methods. The code is available at https://github.com/schwallergroup/augmented_memory.

## Introduction

A quintessential task in any molecular discovery campaign is identifying promising candidate molecules amidst an enormous chemical space.^1^ With the democratization of computing resources, computational oracles can be deployed to query larger chemical spaces in the search of the desired property profile. The use of such oracles has enabled researchers to identify functional materials,^2^ therapeutics,^3−5^ and catalysts,^6^ thus accelerating chemical discovery. However, there is generally a trade-off between oracles, e.g., a computational prediction for binding affinity, predictive accuracy, and inference cost, such that the computational budget imposes a pragmatic constraint. This is exacerbated when the design objective consists of multiple oracles, comprising a multiparameter optimization (MPO) problem that is ubiquitous in molecular design. Correspondingly, designing computational workflows and algorithms that are performant under minimal oracle calls is widely beneficial to the field of molecular design.

**Figure 1:**
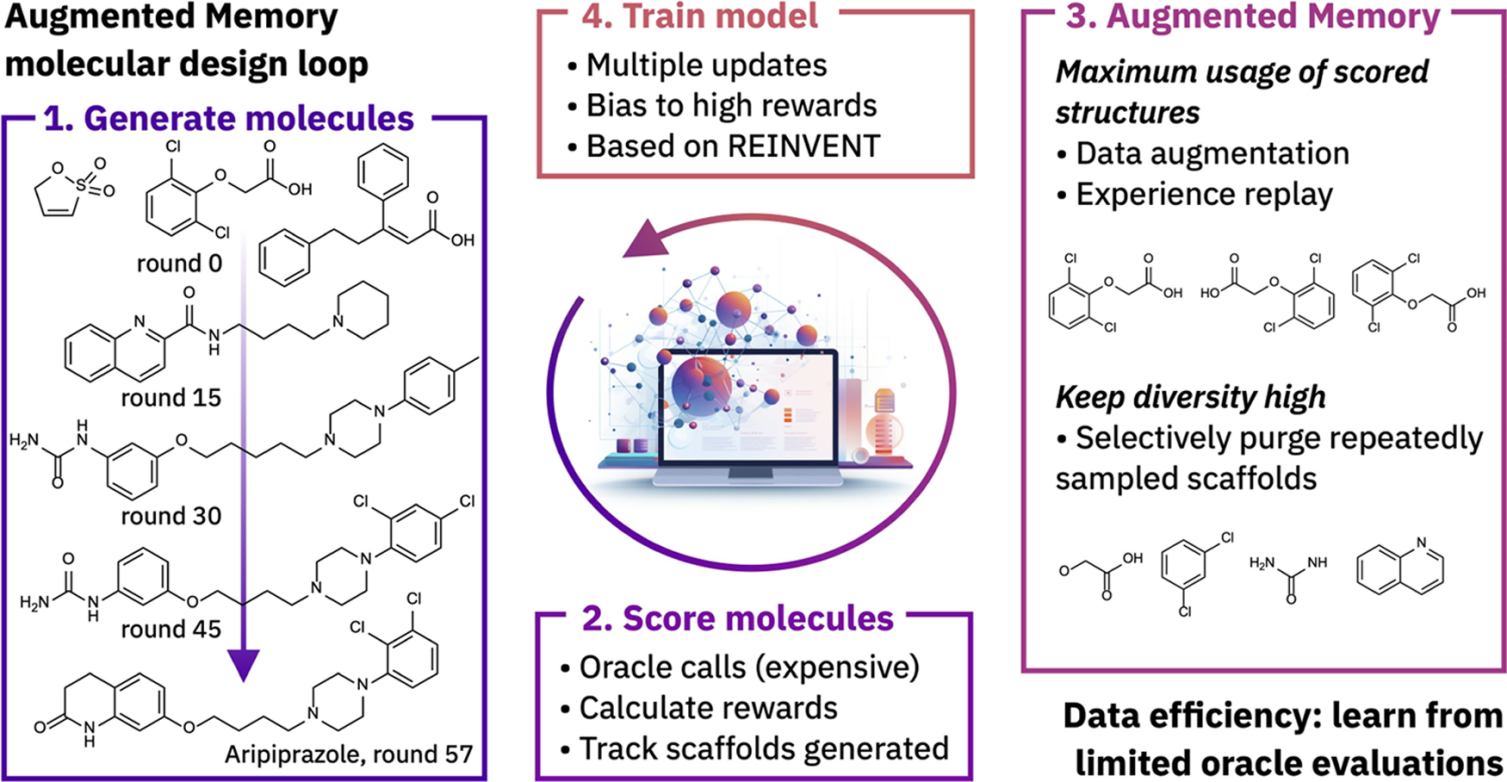
Overview of the sample-efficient Augmented Memory *de novo* molecular design approach.

Recent advancements in *de novo* molecular design have positioned generative methods as a complementary approach to traditional virtual screening.^3,7−10^ Core advantages of these models include the ability to sample chemical space outside the training data and by coupling an optimization algorithm, goal-directed learning can be achieved.^11^ Although the field is relatively nascent, molecular generative models have identified experimentally validated therapeutic molecules^4,5,12−28^ and organocatalysts.^6^ An important shared commonality between these success stories is the inclusion of relatively computationally expensive oracles. In drug design, molecular docking is frequently used, while in catalyst and materials design, quantum-mechanical (QM) properties are of interest. Correspondingly, many generative models proposed in recent years have competed with each other to demonstrate accelerated optimization of these properties. However, the heterogeneity of the assessment protocols makes comparisons difficult. Recently, Gao et al.^29^ proposed the practical molecular optimization (PMO) benchmark, which assesses 25 molecular generative models across 23 tasks, enforcing a computational budget of 10,000 oracle calls. Their results show that REINVENT,^30,31^ a recurrent neural network (RNN)-based generative model operating on simplified molecular-input line-entry system (SMILES)^32^ is, on average, the most sample-efficient generative model. REINVENT^30,31^ uses a policy-based reinforcement learning (RL) algorithm to optimize a reward function in a goal-directed approach. Alternative learning strategies to RL include genetic algorithms (GAs),^33−35^ Bayesian optimization (BO),^36^ and conditional generation.^37−39^ Recently, modifications to REINVENT’s algorithm have been proposed in the form of Best Agent Reminder (BAR)^40^ and Augmented Hill Climbing (AHC),^41^ which both introduce bias toward high-rewarding molecules to improve sample efficiency. Other studies show that experience replay, where the highest rewarding molecules sampled are stored and replayed to the model, improves sample efficiency.^25,31^ More recently, Bjerrum et al.^42^ proposed double-loop RL to take advantage of the noninjective nature of SMILES and the ease with which they can be augmented. By obtaining different SMILES sequences for the same molecule, oracle scores can be reused to perform multiple updates to the Agent. Their results show accelerated learning while maintaining the diversity of results, an aspect missing in many proposed benchmarks. Sample efficiency is a limiting factor to enabling more exploration of chemical spaces of interest, such as in drug discovery where high reward molecules are sparse, i.e., finding a needle in the haystack. In this paper, we highlight the importance of experience replay in policy-based RL algorithms for molecular generation. We propose a novel algorithm called Augmented Memory that combines experience replay with SMILES augmentation (Figure 1). By augmenting the highest rewarding molecules in the replay buffer and using those gradients to update the model, we show that this extreme biasing leads to accelerated learning. However, this base method is susceptible to mode collapse, i.e., the model samples the same molecule repeatedly or becomes stuck at suboptimal minima and is thus unsuitable for use cases requiring exploration of chemical space. To rescue mode collapse, we propose Selective Memory Purge, which removes entries in the replay buffer with chemical scaffolds of which we wish to discourage further sampling of. Applying our strategy, the model is robust against mode collapse despite extremely biased gradients, maintaining accelerated learning and the ability to explore the chemical space, if desired. The translatable advantage of Augmented Memory is its ability to generate molecules optimized for the target property profile with minimal calls to expensive oracles. The main contributions of this paper are

- We propose a novel algorithm called Augmented Memory, which significantly outperforms all previous algorithms in sample efficiency. This is demonstrated in an exploitation task, the PMO benchmark, and in both drug and materials design case studies.
- We propose a method called Selective Memory Purge, which can be used in conjunction with Augmented Memory to generate diverse molecules while retaining enhanced sample efficiency.
- We explicitly highlight the importance of experience replay on the sample efficiency of REINVENT and all proposed algorithmic modifications.
- We expand the PMO benchmark^29^ by adding Augmented Memory and BAR^40^ implementations. We further add experience replay to the implemented version of AHC^41,43^ for comparison.

## Methods

### Goal-Directed Molecular Design with Policy-Based Reinforcement Learning

Molecular generation can be framed as a policy-based RL problem, where a base model (Prior) is trained on a general data set and fine-tuned (Agent) to generate molecules with desired property profiles. Existing works that follow this paradigm include SMILES-based RNNs,^30,31,44,45^ transformers,^37,46−49^ generative adversarial networks (GANs),^50−54^ variational autoencoders (VAEs),^4,55^ graph-based models,^40,56−58^ and GFlowNets.^59^ While all methods can generate valid molecules and the policy can be fine-tuned via RL, none of the previous methods jointly address sample efficiency and a reliable mechanism to mitigate mode collapse. We note that GFlowNets^59^ by construction can achieve diverse sampling but are not as sample-efficient as demonstrated in the PMO benchmark.^29^ By contrast, SMILES-based models, particularly REINVENT,^30,31^ have been shown to be among the most sample-efficient molecular generative models, even when compared to the newest proposed models.

This has been shown in diverse benchmarks, such as GuacaMol,^60^ MOSES,^61^ and PMO.^29^ Our proposed Augmented Memory algorithm builds on this observation and exploits the noninjective nature of SMILES.

### Sample Efficiency in Molecular Design

Many existing policy-based RL works for molecular design operating on SMILES are based on the REINFORCE^62^ algorithm. Alternative formulations operating on Cartesian coordinates often use actor-critic architectures^63,64^ and are based on the Proximal Policy Optimization (PPO)^65^ algorithm. Other existing actor-critic methods have used SMILES-level^66^ or fragment-level encodings.^67,68^ Many algorithmic modifications to SMILES-based models to improve sample efficiency present a unifying theme of using biased gradients to direct the policy toward chemical space with high reward. Neil et al.^69^ explored Hill Climbing (HC) and PPO. Similarly, Atance et al.^40^ introduced Best Agent Reminder (BAR), which keeps track of the best agent and reminds the current policy of favorable actions. Thomas et al.^41^ introduced Augmented Hill Climbing (AHC), a hybrid of HC and REINVENT’s algorithm, which updates the policy at every epoch using only the top-k fraction of generated molecules and shows improved sample efficiency. However, sample efficiency by itself is not sufficient for practical applications of molecular generative models, as one should aim to generate diverse molecules that satisfy the objective function. To address this limitation, Bjerrum et al.^42^ built directly on REINVENT and introduced double-loop RL. By performing SMILES augmentation, the policy can be updated numerous times per oracle call. Their results showed improved sample efficiency compared to AHC while maintaining diverse sampling.

### Experience Replay for Molecular Design

Experience replay was first proposed by Lin et al.^70^ as a mechanism to replay past experiences to the model so that it can learn from the same experience numerous times. Two paradigms in RL are on-policy and off-policy, where the model’s actions are dictated by its current policy or a separate policy known as the behavior policy, respectively.^71^ Experience replay is usually applied in off-policy methods, as past experiences are less likely to be applicable to the current policy. In molecular design, experience replay has been proposed by Blaschke et al.^31,72^ and Korshunova et al.^25^ to keep track of the best molecules sampled so far, based on their corresponding reward. Hu et al.^49^ used a similar formulation and empirically showed its benefit. Notably, these applications of experience replay are for on-policy learning using the REINFORCE algorithm. In contrast, Yang et al.^73^ used prioritized experience replay (PER) in their fragment-based generative model called FREED in the off-policy setting. We note that a similar mechanism was proposed by Putin et al.^52^ using an external memory.

## Augmented Memory

**Figure 2:**
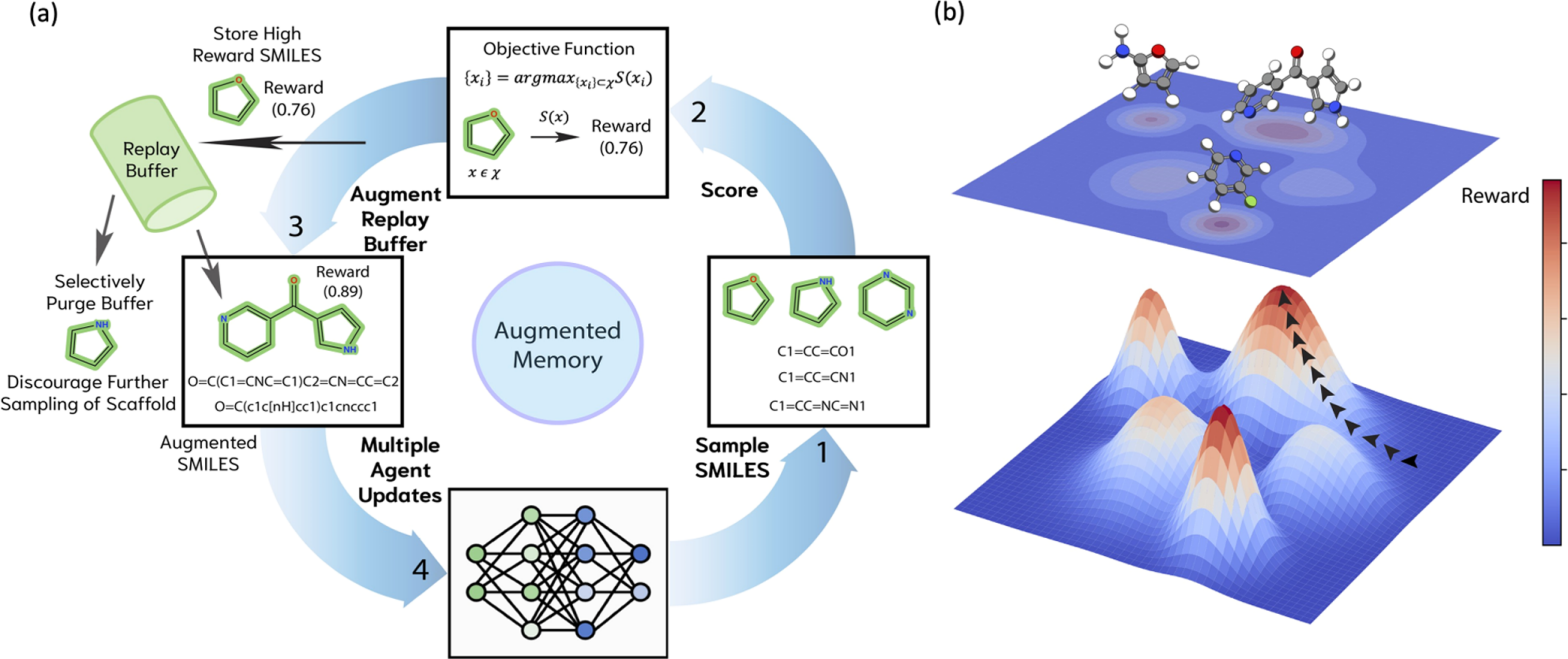
Augmented Memory. (a) The proposed method proceeds via four steps: 1. Generate a batch of SMILES according to the current policy. 2. Compute the reward for the SMILES given the objective function. 3. Update the replay buffer to keep only the top-k molecules. Optionally, remove molecules from the replay buffer to discourage further sampling of specific scaffolds. Perform SMILES augmentation of both the sampled batch and the entire replay buffer. 4. Update the Agent and repeat step 3 *N* times. (b) Schematic of the intended behavior. Augmenting the entire replay buffer and updating the Agent repeatedly direct chemical space exploration to areas of high reward.

In this work, we extend the observations by Blaschke et al.^72^ and Korshunova et al.^25^ and explicitly show the benefit of experience replay for small molecule design in dense reward environments, i.e., most molecules give at least some reward, for on-policy learning given a static objective function. This static nature means that regardless of the current policy, high-rewarding molecules will always receive the same reward, which supports the efficacy of experience replay in the on-policy setting for molecular generation. Next, we combine elements of HC and SMILES augmentation with experience replay and propose to update the policy at every fine-tuning epoch using the entire replay buffer. A reward shaping mechanism^74^ is introduced by using these extremely biased gradients toward high-rewarding chemical space, which we show significantly improves sample efficiency. This section describes each component of Augmented Memory (Figure 2) that is capable of performing MPO.

### Augmented Likelihood Loss

The molecular generative model builds directly on REINVENT^30,31^ and is an autoregressive SMILES-based RNN using long short-term memory (LSTM)^75^ cells. The generative process is cast as an on-policy RL problem by defining the state space, *S*_*t*_, and the action space, *A*_*t*_(*s*_*t*_). Since REINVENT is a language model and samples tokens, *S*_*t*_ denotes every intermediate sequence of tokens leading up to the fully constructed SMILES and *A*_*t*_(*s*_*t*_) is the token sampling probability at every intermediate state. *A*_*t*_(*s*_*t*_) is controlled by the policy, π_θ_, which is parametrized by the RNN. An assumption is that the SMILES generation process is Markovian (eq 1):
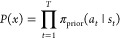
1The Augmented Likelihood is defined as a linear combination between the Prior Likelihood and the scoring function, *S*, which returns a reward denoting the desirability of a given molecule and is modulated by a hyperparameter sigma, σ (eq 2). The Prior Likelihood term acts to ensure that the generated SMILES are syntactically valid and has been shown to empirically enforce reasonable chemistry.^30,41^


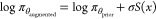
2Maximizing the expected reward is equivalent to minimizing the squared difference (eq 3) between the Augmented Likelihood and the Agent Likelihood (derivation in the Supporting Information). The full pseudocode for the algorithm is shown in the Supporting Information.
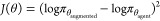
3

### SMILES Augmentation

SMILES^32^ are noninjective and yield different sequence representations given a different atom numbering in the molecular graph, i.e., augmented SMILES. In this work, SMILES are generated by performing a depth-first search (DFS) of the molecular graph using RDKit.^76^ By shuffling the atom numbering and hence starting the DFS at different atoms, augmented SMILES sequences can be generated.^77^ SMILES-based molecular generative models have taken advantage of this to train performant models under low-data regimes, e.g., by artificially increasing the data set size via data augmentation,^78^ and to increase chemical space generalizability^77^ by training a Prior model on augmented SMILES. Similar to Bjerrum et al.,^42^ we reuse scores obtained from the oracle to update the Agent multiple times by passing different augmented SMILES representations.

### Experience Replay

Experience replay is implemented in REINVENT as a buffer that stores a predefined maximum number of the highest rewarding SMILES sampled so far (100 in this work). Usually, during each sampling, a subset of the buffer is replayed to the Agent.^31^ In our proposed method, all SMILES in the buffer are augmented, and using their corresponding reward, the Agent is updated multiple times according to the loss function given in eq 3.

### Selective Memory Purge

Blaschke et al.^72^ introduced memory-assisted RL to enforce diverse sampling in REINVENT via diversity filters (DFs). During the generative process, the scaffolds of sampled molecules are stored in “buckets” with predefined and limited size. Once a bucket has been fully populated, further sampling of the same scaffold results in zero reward. We incorporate this heuristic to enforce diversity in our proposed method called Selective Memory Purge. At every epoch, the replay buffer is purged of any scaffolds that are penalized by the DF. In this work, we define scaffolds as Bemis–Murcko scaffolds,^79^ which consider heavy atoms. The effect is that each augmentation round only updates the Agent with scaffolds that still receive reward, preventing the Agent from becoming myopic and leading to suboptimal convergence.

## Results and Discussion

We designed four experiments to assess our method. First, we explicitly demonstrate the importance of experience replay and identify optimal parameters for Augmented Memory using the aripiprazole similarity experiment. Next, we benchmark its performance on the practical molecular optimization (PMO)^29^ benchmark containing 23 tasks. We demonstrate the practical applicability of our method in a dopamine type 2 receptor (DRD2) drug discovery case study. Lastly, we extend Augmented Memory to a functional materials design case study, optimizing explicitly for QM properties computed using xTB.^80^ The Supporting Information includes details on the data set, hyperparameters, and ablation studies.

## Aripiprazole Similarity

The aripiprazole similarity task is from the GuacaMol benchmark^60^ and the objective is to successfully sample aripiprazole. This experiment was used to demonstrate the importance of experience replay in dense reward environments and compare Augmented Memory to existing policy-based algorithms proposed for molecular generative models, which introduced biased gradients, including BAR^40^ and AHC.^41^ We also compare to double-loop RL as proposed by Bjerrum et al.^42^ since both methods use SMILES augmentation. As the code for double-loop RL was not released, we took the values reported in their paper, which holds as the method was also built directly on REINVENT,^31^ uses the same pretrained Prior, and hyperparameters. Moreover, in the studies presenting AHC^41^ and BAR,^40^ experience replay was not used but we provide an implementation and further compare their performance. The hyperparameters used for all models were kept default and are presented in the Supporting Information.

### Experience Replay is Vital for Sample Efficiency

**Figure 3:**
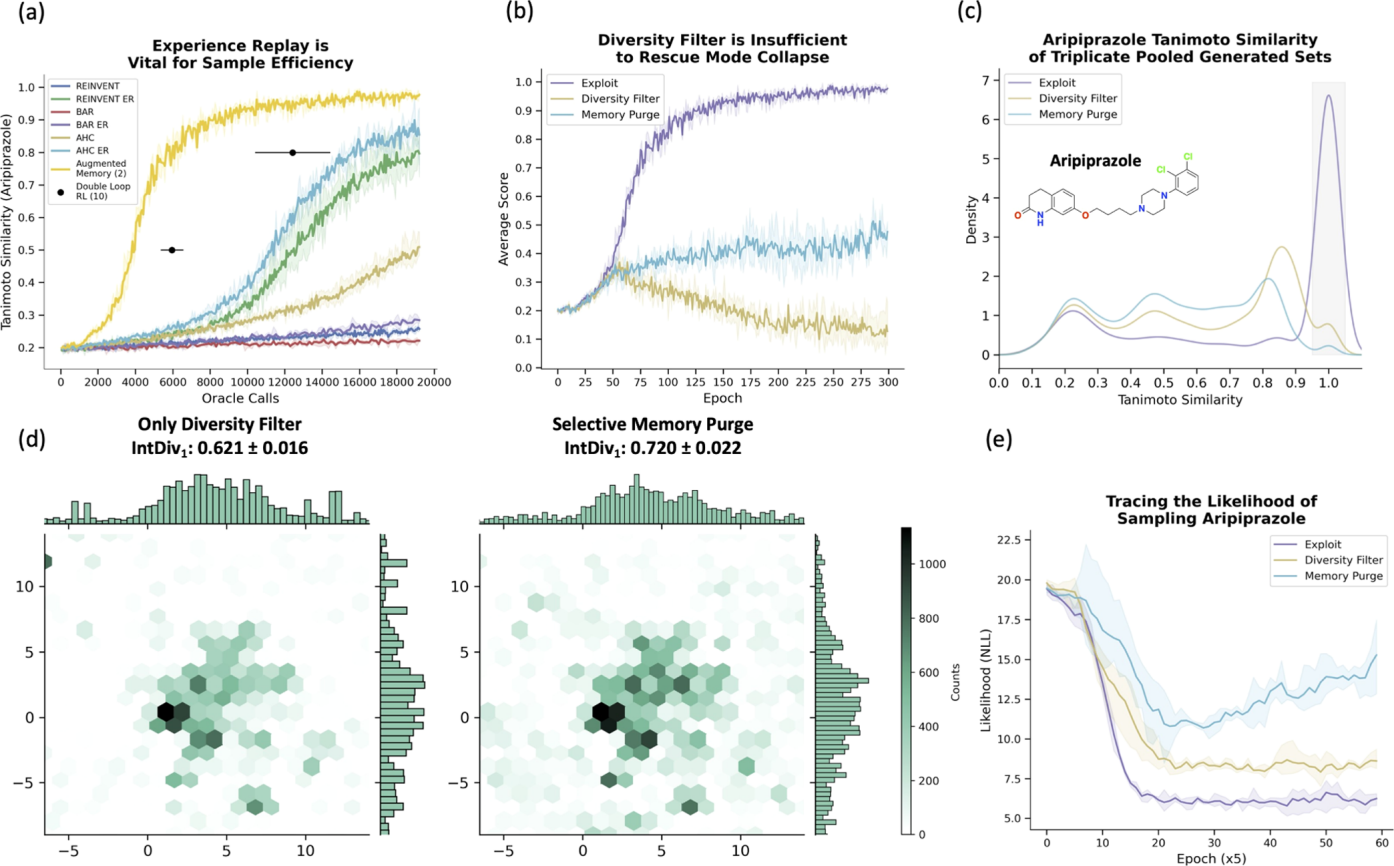
Augmented Memory and Selective Memory Purge significantly improve the sample efficiency and enable diverse sampling. The shaded region represents the minimum and maximum scores across triplicate runs. (a) Comparing sample efficiency of on-policy algorithms. Experience replay (ER) improves all base algorithms. The values for double-loop RL^42^ are taken from the original paper as the code is not released. The black dots are the mean at 0.5 and 0.8 and the standard deviation across triplicate runs. (b) Average score for aripiprazole similarity. In the Diversity Filter and Memory Purge experiments, scores of 0 are given if the Agent repeatedly samples the same scaffold. (c) Pooled Tanimoto similarities. Memory purge rediscovers aripiprazole and has a flatter distribution, suggesting increased exploration. (d) UMAP^81^ and IntDiv1^61^ metrics showing qualitatively and quantitatively increased exploration using Memory Purge. The plots were generated using ChemCharts.^82^ (e) The negative log-likelihood of sampling aripiprazole across the full generative experiments.

We demonstrate that experience replay significantly improves sample efficiency in dense reward environments (Figure 3). We first identified the optimal number of augmentation rounds for Augmented Memory as two for training stability. Increasing the number of augmentation rounds can further improve sample efficiency but can lead to mode collapse (see the Supporting Information). Next, we compare baseline RL (original implementation of REINVENT^30,31^), AHC,^41^ BAR,^40^ and double-loop RL^42^ with our method. Augmented Memory significantly outperforms all other algorithms and reaches a score of 0.8 with 6,144 oracle calls (average over 100 replicates). Double-loop RL^42^ uses experience replay and is the second-most sample-efficient algorithm and reaches a score of 0.8 after 12,416 ± 1984 oracle calls (as stated in their paper), which is twice the number of oracle calls required compared to our method. Moreover, the key observation we convey is that experience replay improves upon the base algorithm in all cases (Figure 3). For example, AHC^41^ with the newly implemented experience replay reaches a score of 0.8 but with more than 2.5× the oracle calls (15,616). Our observations around experience replay are supported by previous works.^25,31^ Finally, we show that augmentation is crucial for enhanced sample efficiency in the Supporting Information.

### Selective Memory Purge Enables Diverse Sampling while Retaining Efficiency

Figure 3 demonstrates the enhanced sample efficiency of Augmented Memory, but real-world applications of molecular generative models require the ability to sample diverse solutions. While aripiprazole is inherently an exploitation task, it can be framed as an exploration task if the goal is rephrased as rediscovering the target molecule and generating similar molecules. Using this formulation, we design experiments to prove that Augmented Memory can achieve diverse sampling. Figure 3 shows the training plot across three methods: pure exploitation where diversity is not enforced, exploration using a diversity filter (DF),^72^ and Selective Memory Purge. In the pure exploitation scenario, aripiprazole is rediscovered quickly (score of 1.0). In the DF experiment, where a score of 0 is assigned for scaffolds sampled more than 25 times, mode collapse is observed (Figure 3). By contrast, Selective Memory Purge maintains a moderate average score. The results from triplicate experiments were pooled to investigate the density of aripiprazole similarities (Figure 3). As expected, in the pure exploitation scenario, most molecules are aripiprazole (Tanimoto similarity of 1.0). DF and Selective Memory Purge both enforce a wider distribution of similarities but to varying degrees. In the shaded region (rediscovery score), Selective Memory Purge shows only a small density relative to DF. Moreover, Selective Memory Purge shows a flatter distribution of similarities. These observations demonstrate that Selective Memory Purge rediscovers the target molecule and enforces increased exploration compared to DF. To investigate this further, the same pooled data set was embedded using Uniform Manifold Approximation and Projection (UMAP)^81^ to visualize the chemical space. Qualitatively and quantitatively, the Selective Memory Purge covers a larger chemical space (Figure 3). The internal diversity (IntDiv1) metric was calculated as proposed in the MOSES benchmark^61^ and measured the diversity within a set of generated molecules. Finally, we save the Agent states at every 5 epochs across the entire generative run and trace the negative log-likelihood (NLL) of sampling aripiprazole (Figure 3). It is evident that Selective Memory Purge can discourage sampling of the target molecule more effectively than using only a DF. Importantly, the NLL also diverges, suggesting that the Agent is increasingly moving to chemical space dissimilar to that of aripiprazole as the generative experiment progresses.

## Practical Molecular Optimization (PMO) Benchmark

The main motivation of our method is to improve the sample efficiency. This would enable molecular generative models to explicitly design molecules satisfying more expensive oracles with increased predictive accuracy. We benchmark our method on the PMO benchmark proposed by Gao et al.,^29^ which restricts the number of oracle calls to 10,000 and encompasses 23 tasks. The metric used is the area under the curve (AUC) for the top 10 molecules. We note that Thomas et al.^43^ proposed a modified AUC Top-10 metric that incorporates diversity, but we omit comparison as the formulation can be subjective. The current Top AUC-10 metric assesses sample efficiency, which is our focus. In the original PMO paper, REINVENT,^30^ which natively uses experience replay, is the most sample-efficient model. We compare our method directly to REINVENT, BAR,^40^ and AHC,^41^ which reports improved sample efficiency compared to REINVENT and is open-sourced. We also add experience replay to BAR and AHC to further highlight its importance for sample efficiency. For a more statistically convincing comparison, we perform 10 independent runs (using 10 different seeds) compared to 5 used in the original PMO paper as the authors benchmarked 25 models, which imposed a significant computational cost. The optimal hyperparameters for REINVENT and AHC were used as provided in the PMO repository. We perform hyperparameter optimization for BAR following the PMO protocol (see the Supporting Information), and Augmented Memory was run using REINVENT’s optimal hyperparameters. The results show that Augmented Memory outperforms all methods (Table 1) and achieves superior performance to REINVENT across 14/23 benchmark tasks (statistically significant at the 95% confidence level). Moreover, the results reinforce the importance of experience replay as it improves the sample efficiency of both BAR and AHC, although neither outperforms REINVENT. Finally, in the PMO paper,^29^ models were ranked based on the sum of the total AUC Top-10 and adjacently ranked models typically differ by 0.3–0.5. Augmented Memory outperforms REINVENT by 0.986 AUC Top-10 and yields a new state-of-the-art performance on the PMO benchmark.

**Table 1:** Performance of Augmented Memory, REINVENT,^30,31^ AHC,^41^ and BAR^40^ on the PMO Benchmark^a^,^29^

benchmark task	Augmented Memory	REINVENT	AHC replay	BAR replay	AHC	BAR
albuterol_similarity	0.913 ± 0.009	0.871 ± 0.031	0.792 ± 0.030	0.715 ± 0.031	0.745 ± 0.024	0.654 ± 0.026
amlodipine_mpo	0.691 ± 0.047	0.657 ± 0.025	0.596 ± 0.023	0.551 ± 0.01	0.578 ± 0.012	0.533 ± 0.006
celecoxib_rediscovery	0.796 ± 0.008	0.717 ± 0.048	0.697 ± 0.029	0.574 ± 0.025	0.583 ± 0.070	0.452 ± 0.023
deco_hop	0.658 ± 0.024	0.672 ± 0.052	0.650 ± 0.030	0.596 ± 0.006	0.632 ± 0.032	0.586 ± 0.003
drd2	0.963 ± 0.006	0.939 ± 0.012	0.913 ± 0.011	0.910 ± 0.018	0.912 ± 0.009	0.893 ± 0.052
fexofenadine_mpo	0.859 ± 0.009	0.783 ± 0.021	0.747 ± 0.004	0.711 ± 0.006	0.749 ± 0.005	0.700 ± 0.008
gsk3b	0.881 ± 0.021	0.870 ± 0.026	0.819 ± 0.025	0.722 ± 0.038	0.800 ± 0.021	0.673 ± 0.049
isomers_c7h8n2o2	0.853 ± 0.087	0.856 ± 0.042	0.682 ± 0.037	0.708 ± 0.197	0.631 ± 0.084	0.740 ± 0.082
isomers_c9h10n2o2pf2cl	0.736 ± 0.051	0.641 ± 0.038	0.276 ± 0.133	0.618 ± 0.049	0.191 ± 0.096	0.529 ± 0.033
jnk3	0.739 ± 0.110	0.723 ± 0.147	0.649 ± 0.056	0.559 ± 0.047	0.616 ± 0.092	0.457 ± 0.118
median1	0.326 ± 0.013	0.368 ± 0.011	0.346 ± 0.008	0.285 ± 0.007	0.338 ± 0.014	0.269 ± 0.011
median2	0.291 ± 0.008	0.279 ± 0.005	0.273 ± 0.005	0.227 ± 0.009	0.265 ± 0.005	0.201 ± 0.005
mestranol_similarity	0.750 ± 0.049	0.637 ± 0.041	0.599 ± 0.031	0.486 ± 0.015	0.561 ± 0.022	0.456 ± 0.018
osimertinib_mpo	0.855 ± 0.004	0.836 ± 0.007	0.810 ± 0.003	0.799 ± 0.003	0.809 ± 0.002	0.793 ± 0.005
perindopril_mpo	0.613 ± 0.015	0.561 ± 0.019	0.487 ± 0.012	0.470 ± 0.007	0.482 ± 0.008	0.457 ± 0.009
qed	0.942 ± 0.000	0.941 ± 0.000	0.941 ± 0.000	0.941 ± 0.000	0.941 ± 0.000	0.939 ± 0.001
ranolazine_mpo	0.801 ± 0.006	0.768 ± 0.008	0.721 ± 0.00	0.710 ± 0.014	0.722 ± 0.008	0.708 ± 0.012
scaffold_hop	0.567 ± 0.008	0.556 ± 0.019	0.535 ± 0.007	0.486 ± 0.005	0.525 ± 0.008	0.467 ± 0.005
sitagliptin_mpo	0.284 ± 0.050	0.049 ± 0.067	0.022 ± 0.008	0.182 ± 0.033	0.028 ± 0.011	0.107 ± 0.034
thiothixene_rediscovery	0.550 ± 0.041	0.531 ± 0.036	0.519 ± 0.012	0.401 ± 0.016	0.467 ± 0.032	0.356 ± 0.010
troglitazone_rediscovery	0.540 ± 0.048	0.428 ± 0.028	0.409 ± 0.020	0.312 ± 0.008	0.371 ± 0.019	0.282 ± 0.010
valsartan_smarts	0.000 ± 0.000	0.091 ± 0.273	0.000 ± 0.000	0.000 ± 0.000	0.000 ± 0.000	0.000 ± 0.000
zaleplon_mpo	0.394 ± 0.026	0.269 ± 0.083	0.072 ± 0.032	0.315 ± 0.040	0.047 ± 0.013	0.291 ± 0.026
sum of AUC Top-10 (↑)	**15.002**	14.016	12.555	12.278	11.993	11.543
PMO rank (*n*/30)	**1**	2	7	8	11	12

a The mean and standard deviation of the AUC Top-10 is reported. The values obtained for REINVENT differ slightly from the PMO paper, as we performed 10 independent runs compared to 5. Superior performance to REINVENT is bolded (statistically significant based on *t*-tests at the 95% confidence level).

## Dopamine Type 2 Receptor (DRD2) Case Study

**Figure 4:**
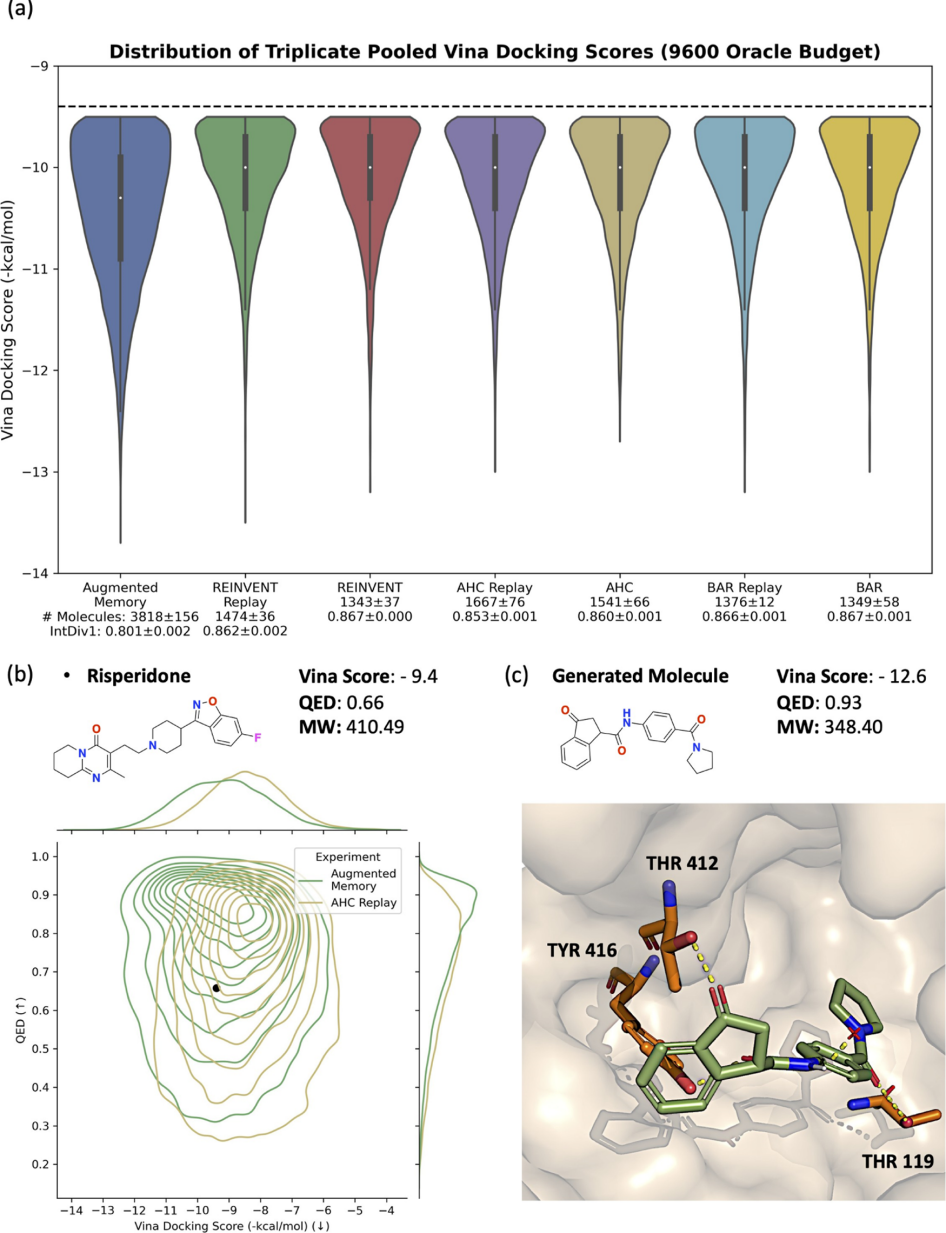
DRD2 case study. PDB ID: 6CM4. (a) Docking score distribution of all compared algorithms. (b) Augmented Memory jointly optimizes QED-Vina score, demonstrating the ability to perform MPO. (c) Binding pose of a generated molecule using Augmented Memory. The three components in the objective function: MW < 500, QED, and Vina docking score are all optimized.

To prove that Augmented Memory can perform MPO, we formulate a case study to generate potential dopamine type 2 receptor (DRD2) inhibitors^83^ by explicitly optimizing molecular docking scores (Figure 4). For accessibility and reproducibility, we use the open-source AutoDock Vina^84^ for docking. A well-known failure mode of docking algorithms is that they reward lipophilic molecules, e.g., possessing many carbon atoms, which can be promiscuous binders.^85,86^ Bjerrum et al.^42^ considered this and enforced molecules to possess a molecular weight (MW) < 500 Da, but this is insufficient in preventing exploitation of the docking algorithm as we show in the Supporting Information. Following Guo et al.,^87^ we design the MPO as follows: MW < 500 Da, maximize QED,^88^ and minimize the Vina docking score, for chemical plausibility. AutoDock Vina is a relatively expensive oracle and we impose a computational budget of 9600 oracle calls, similar to the 10,000 oracle calls enforced in the PMO^29^ benchmark. We compare Augmented Memory, REINVENT,^30,31^ AHC,^41^ and BAR^40^ as the optimization algorithms. To mimic a real-world drug discovery pipeline that discards unpromising molecules, we pool the results from triplicate experiments with the following filter: MW < 500 Da, QED > 0.4 (the DRD2 drug molecule, risperidone, has a QED of 0.66), and Vina docking score < −9.4 (risperidone’s score). Figure 4 shows the docking score distribution with the number of molecules passing the filter and the IntDiv1^61^ score annotated. First, experience replay improves all base algorithms, further reinforcing its importance. Second, all algorithms with the exception of Augmented Memory perform similarly. Compared to AHC with experience replay, which is the second-most sample-efficient algorithm, Augmented Memory generates over 2000 more molecules with a better docking score than risperidone, with a small trade-off in diversity (IntDiv1 of 0.801). We emphasize that AHC with experience replay does not even generate 2000 molecules passing the filter. To further prove the optimization capability, Figure 4 shows a contour plot of the QED-Vina score distribution for Augmented Memory and AHC with experience replay. It is clear that the joint QED-Vina score distribution for Augmented Memory is shifted to higher QED values and lower Vina scores. The black dot is risperidone, and the bulk density of AHC does not possess a better docking score. In the Supporting Information, we ran this same experiment with twice the oracle budget (19,200 calls) to show that the benefits of Augmented Memory are retained. With this increased budget, Augmented Memory can still sometimes find more than three times the number of molecules passing the filter compared to the other algorithms. Finally, Figure 4 shows an example binding pose of a molecule generated using Augmented Memory. We highlight that the chemical plausibility of the structure is enforced precisely because MW and QED are also included in the MPO objective, thus representing a more realistic case study.

## Generated Molecules: Plausibility of Binding Poses

**Figure 5:**
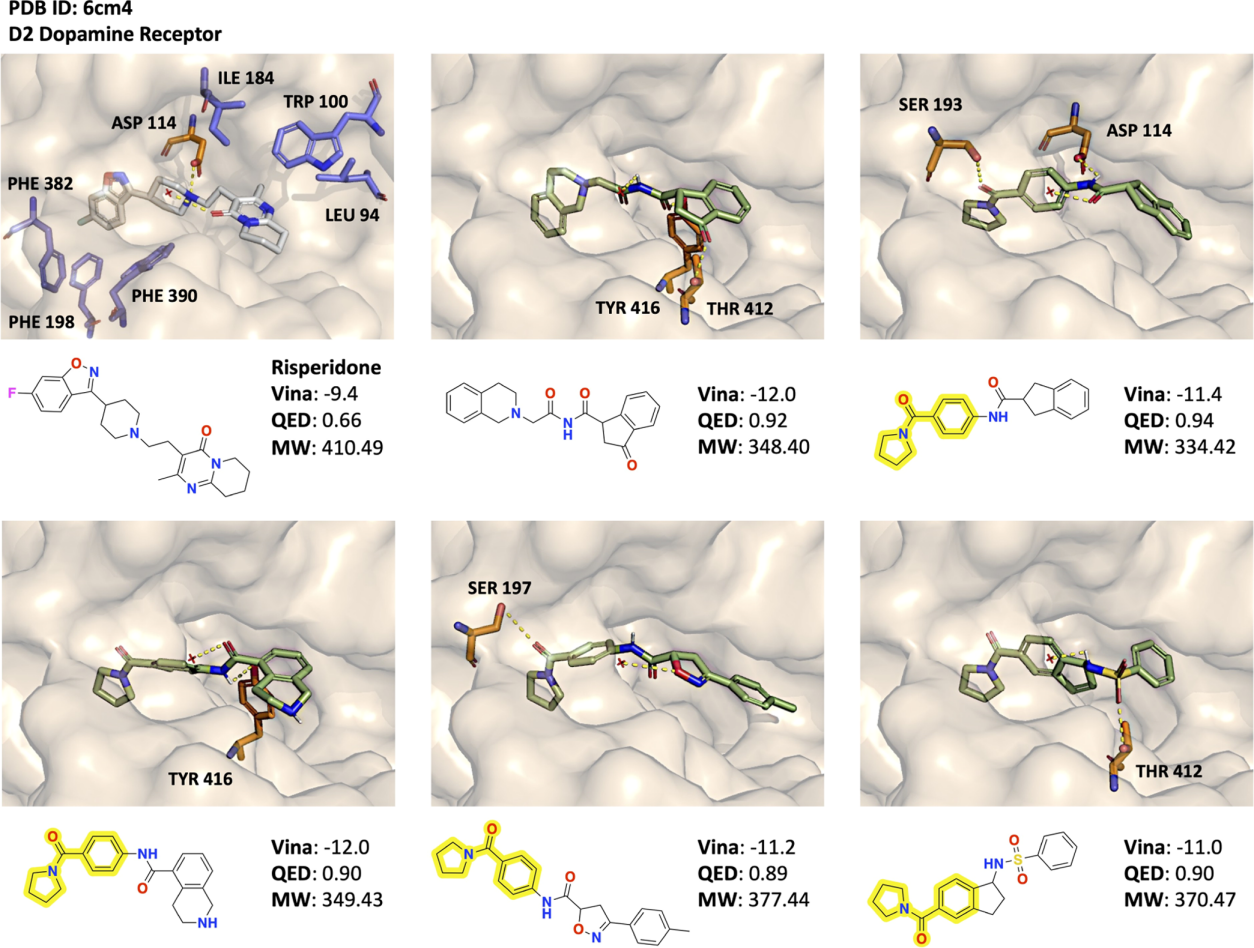
More examples of DRD2 (PDB ID: 6CM4) binding poses. Reference drug molecule risperidone contrasted with generated molecules from Augmented Memory (randomly selected among the highest rewarding molecules). The yellow highlighted 1-benzoylpyrrolidine substructure is common among several generated molecules. Residues involved in hydrogen-bonding interactions are shown in orange. Residues involved in hydrophobic interactions are shown in blue. Yellow-dotted lines indicate specific hydrogen-bonding interactions with receptor residues (annotated). The red asterisk is water.

Next, we assess additional binding poses of high reward molecules generated by Augmented Memory. To do so, we first draw insights from Wang et al.,^83^ who elucidate the structure of DRD2 bound to the inverse agonist, risperidone. Risperidone binds to DRD2 through a combination of hydrophobic and hydrogen-bonding interactions. The fluoro-benzisoxazole moiety sits in the “hydrophobic pocket” (left side of the binding cavity in Figure 5 poses), and the tertiary amine in the middle forms a salt bridge with Asp 114. Furthermore, through mutation studies, Wang et al.^83^ determined notable residues that are crucial to the binding affinity of risperidone, including Asp 114, Thr 412, and Tyr 416. These residues, when mutated, decrease the binding affinity of risperidone by more than 10-fold.^83^ Cross-referencing the binding poses of generated molecules (Figure 5), many are predicted to form hydrogen-bond interactions with these key residues. Another notable interaction formed by risperidone involves Ser 197 in the hydrophobic pocket, which is retained by one of the shown binding poses (Figure 5). Analyzing the structures of the generated molecules further reveals a common 1-benzoylpyrrolidine substructure (yellow highlighted in Figure 5). In all four binding poses, the 1-benzoylpyrrolidine moiety sits deep in the “hydrophobic pocket”. In this region, there are three key phenylalanine residues (Phe 382, 198, 390) containing a phenyl side chain that risperidone forms hydrophobic interactions with risperidone binding pose in Figure 5.^83^ In the risperidone binding pose (Figure 5), this is facilitated by the fluoro-benzisoxazole containing the benzene ring. Similarly, in the four binding poses of generated molecules with 1-benzoylpyrrolidine, the pyrrolidine (5-membered ring with a nitrogen atom) can also facilitate these hydrophobic interactions. Moreover, the amide linkage of 1-benzoylpyrrolidine is often predicted to form hydrogen-bonding interactions with serine residues, which are also important for risperidone.^83^ In the generated molecule without 1-benzoylpyrrolidine, the bicyclic moiety features a benzene ring in a location and geometry similar to those of risperidone (Figure 5) and thus should also facilitate hydrophobic interactions with the phenylalanine residues. Wang et al.^83^ further performed kinetic studies to elucidate the binding dynamics of risperidone. One key finding is that the series of amino acids toward the entrance of the binding cavity forms a “hydrophobic patch” (consisting of Ile 184, Trp 100, and Leu 94), in which the hydrophobic interactions of risperidone’s tetrahydropyridopyrimidinone facilitate slow dissociation.^83^ Cross-referencing all generated examples, nonpolar ring systems occupy this space and can conceivably also engage in the same interactions. Overall, the molecules have plausible binding poses and retain key interactions formed by risperidone.

## Optoelectronics Case Study: Designing Out-of-Distribution

**Figure 6:**
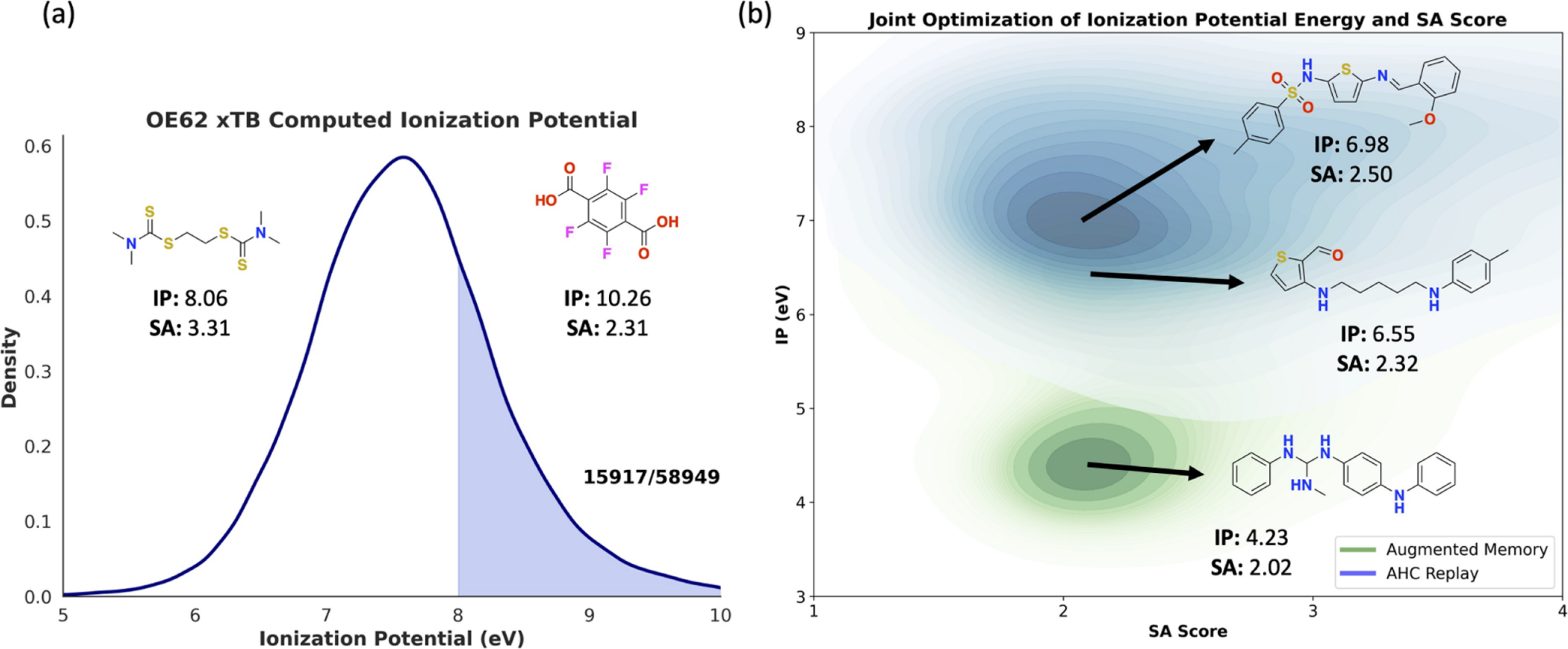
Augmented Memory optimization of xTB quantum mechanical properties. (a) OE62 data set with recomputed ionization potential (IP) energy using xTB. The fraction of the converged data set with IP > 8 eV is shaded, and examples of these molecules are shown. (b) Joint optimization of synthetic accessibility (SA) score and IP energy using Augmented Memory and Augmented Hill Climbing with experience replay with a 5000 oracle call budget. The pooled molecules across triplicate runs are shown. Augmented Memory notably shifts the distribution of molecules to lower SA score and IP energy.

As Augmented Memory is a general optimization algorithm, we extend its applicability to generate molecules optimized for QM properties. Existing works to design molecules tailored for QM properties often leverage a surrogate model trained on DFT calculations, as on-the-fly DFT calculations are too costly. The trade-off to avoiding the costly simulation is that the generative design is constrained to the surrogate model’s domain of applicability, i.e., if the generative model proposes a molecule too dissimilar to the surrogate’s training data, its prediction will more likely be inaccurate. Such workflows have been applied to design optoelectronics^89,90^ and semiconducting materials.^91,92^ By contrast, Li et al.^90^ used REINVENT^30,31^ to design optoelectronics by explicitly optimizing for xTB^80^ and DFT-computed properties, thus mitigating surrogate out-of-domain concerns. Recently, Westermayr et al.^2^ used the OE62^93^ data set and designed optoelectronics materials with out-of-distribution (to the OE62 training data) ionization potential (IP) energy by iteratively performing transfer learning on the G-SchNet^94^ generative model. Inspired by this case study, we design an experiment to showcase Augmented Memory’s ability to shift molecular distributions under minimal oracle calls. The workflow is as follows: Recompute the IP energy of the entire OE62 data set using xTB.^80^ OE62 contains 61,489 molecules, of which 58,949 geometries converged and allowed computing the IP energy (Figure 6). We intentionally keep the molecules with IP energy >8 eV, resulting in a training set of 15,917. To have a data set sufficient for pretraining, we augmented each SMILES ten times. From this pretrained model, we show that Augmented Memory can jointly optimize the synthetic accessibility (SA)^95^ and minimize IP energy under 5000 oracle calls (xTB computations). We compare Augmented Memory to AHC with experience replay, as it is the second-most sample-efficient algorithm based on the drug discovery case study. Figure 6 shows the distribution of all molecules generated across triplicate experiments with MW < 500 Da. Despite the base model being trained on only examples with IP energy >8 eV, Augmented Memory is able to completely shift the distribution compared to AHC with experience replay under 5000 oracle calls. Quantitatively (pooled across triplicate runs), Augmented Memory generates 3832 molecules with IP energy <5 eV compared to 104 for AHC with experience replay. Finally, a qualitative inspection of the molecules shows that lower IP energy is marked by an increased presence of electron-donating groups, which is supported by previous work.^96^ While looking at the presence of certain chemical moieties is often insufficient to justify the observed property values, we emphasize that the purpose of this case study is to show that even in an extreme out-of-distribution learning case study, Augmented Memory can explicitly optimize for QM properties directly acquired through xTB calculations in 5000 oracle calls. Further validation of generated molecules would require DFT calculations as performed by Westermayr et al.^2^

## Conclusions

In this work, we propose Augmented Memory to improve sample efficiency in molecular generative models. We explicitly show that experience replay is vital in dense reward environments. Augmented Memory capitalizes on this observation and applies SMILES augmentation to the replay buffer to update the Agent multiple times per oracle call. Compared with existing algorithms, Augmented Memory significantly improves sample efficiency and is able to sample diverse solutions using the newly proposed Selective Memory Purge heuristic. We benchmark Augmented Memory on the PMO benchmark^29^ and achieve a new state-of-the-art performance, outperforming the previous state-of-the-art on 14/23 tasks (statistically significant at the 95% confidence level) and by a total sum of 0.986 AUC Top-10. Next, we show the practical application of Augmented Memory by mimicking a more realistic drug discovery task. Our method significantly outperforms existing algorithms, as assessed by the property profile of the generated molecules, and can perform MPO. Analysis of the binding poses of generated molecules shows that they retain key interactions of a known drug molecule and possess structural features that are complementary to the binding cavity. We further extend Augmented Memory’s capabilities to generate molecules optimized for quantum-mechanical properties. Specifically, under minimal oracle calls, Augmented Memory can completely shift the distribution of molecules to jointly optimize the synthetic accessibility score and minimize ionization potential energy. Augmented Memory is thus a general optimization algorithm with broad applicability to drug discovery and materials design. However, limitations exist in the interpretability of the model, as it is not straightforward to elucidate *why* certain molecules are generated. One way to probe interpretability is by posthoc analysis of the generated set to look for common substructures, such as in the analysis of the DRD2 inhibitors in Figure 5. Alternatively, one could enforce the generation process to be conditioned on the presence of commonly observed substructures, as shown in recent work.^97^ Moreover, while we have shown that Augmented Memory can generate molecules optimized for quantum-mechanical properties, metal-containing complexes are ubiquitous in the realm of materials design, e.g., catalysts, and SMILES-based representations often do not adequately represent metals. Future work will aim to extend Augmented Memory to other data representations and model architectures. This work also opens up future integration of Augmented Memory with curriculum learning^98^ and active learning^99^ to enable the use of more expensive oracles given a limited computational budget and further provides insights into experience replay for molecular generative models.

## Supporting Information Available

Ablation experiments with insights into the design decisions during algorithm development, docking protocol details, and further details on the Augmented Memory algorithm (PDF)
