# Supplementary material for: Augmented Memory: Sample-Efficient Generative Molecular Design with Reinforcement Learning
Source: JACS Au. 2024 Apr 10;4(6):2160–72. doi: 10.1021/jacsau.4c00066 (PMC11200228; doi:10.1021/jacsau.4c00066)
Supplement: Supplementary file 1 — au4c00066_si_002.pdf [file au4c00066_si_002.pdf]

Augmented Memory: Sample Efficient Generative Molecular Design with Reinforcement Learning Supporting Information

Jeff Guo[†,‡] and Philippe Schwaller[*,†,‡]

†*Laboratory of Artificial Chemical Intelligence (LIAC), Institut des Sciences et Ingénierie Chimiques,*

Ecole Polytechnique Fédérale de Lausanne (EPFL), Lausanne, Switzerland

‡*National Centre of Competence in Research (NCCR) Catalysis,*

Ecole Polytechnique Fédérale de Lausanne (EPFL), Lausanne, Switzerland

E-mail: {jeff.guo,philippe.schwaller}@epfl.ch

The Supporting Information contains further experiments, ablation studies, experiment hyperparameters, and algorithmic details. The code is available at `https://github.com/schwallergroup/augmented_memory`.

Tolerability to Augmentation Rounds

Similar to Esben et al.[1] in their proposed Double Loop RL algorithm, increasing the number of augmentation rounds increases susceptibility to mode collapse (Figure 1). We used the Aripiprazole Similarity task to perform a grid optimization and found two rounds to be optimal for stability. At three rounds, mode collapse is already observed with triplicate runs.

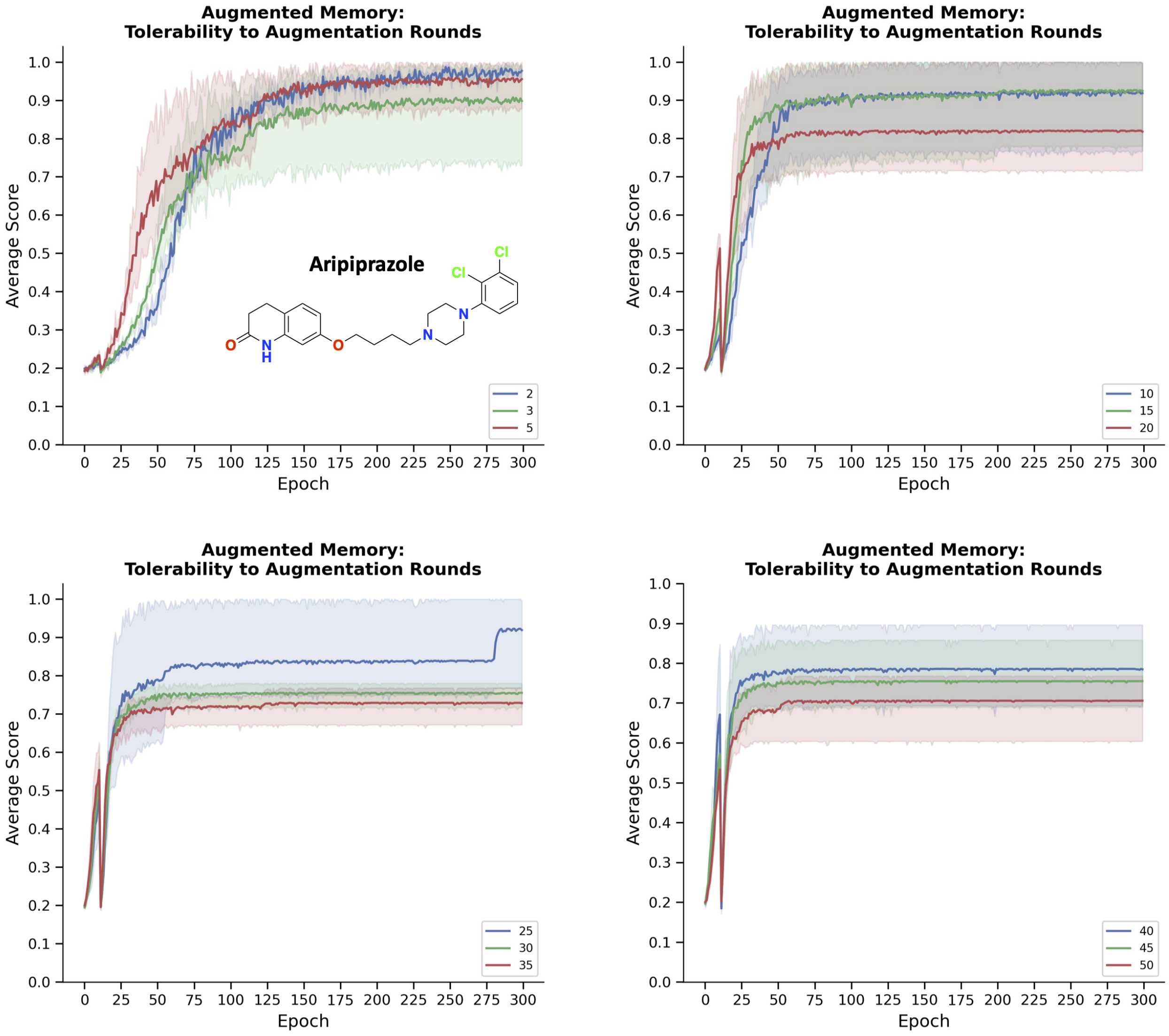

Figure 1: Identifying the optimal augmentation rounds using Aripiprazole Similarity. The shaded region represents the minimum and maximum scores across triplicate runs.

Selective Memory Purge Retains Diversity with Increased Oracle Budget

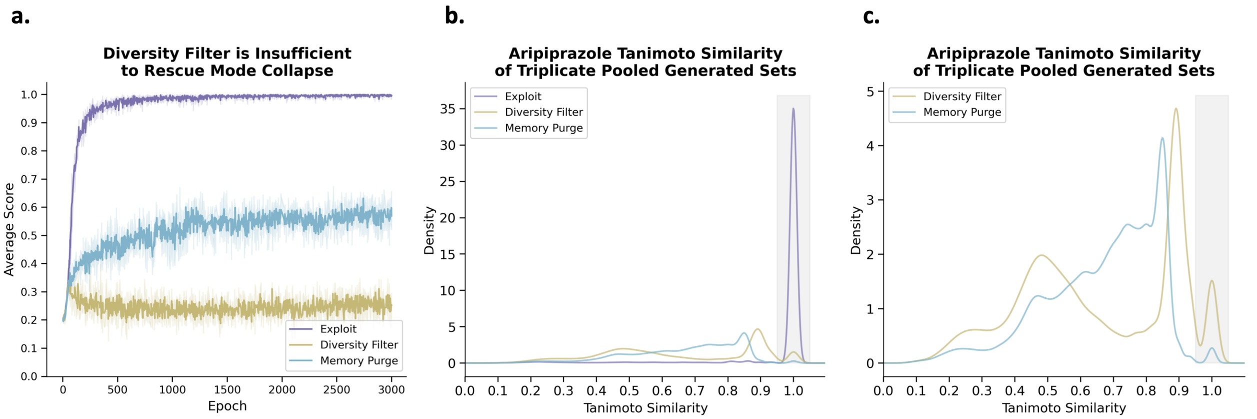

Figure 2: Aripiprazole similarity experiment with ten times the oracle budget. Selective Memory Purge retains the ability to sample diverse molecules, in contrast to using just a Diversity Filter. a. Average Score curve. b. Pooled Tanimoto (triplicate runs) similarities. c. Pooled Tanimoto similarities (triplicate runs) focusing only on Selective Memory Purge and Diversity Filter. Selective Memory Purge generates a broader distribution of high reward molecules.

This section supplements Figure 3b results in the main text and shows that Selective Memory Purge enables Augmented Memory to generate diverse molecules even with a much larger oracle budget (ten times more than the main text). Exactly like the main text, the experiment is to generate Aripiprazole and other high Tanimoto similarity molecules. The use of a Diversity Filter assigns generated molecules a reward of 0 if the same Bemis-Murcko[2] scaffold has been sampled more than 25 times. When using just the Diversity Filter, the Average Score (reward) is low, indicating the same scaffolds are repeatedly sampled (Fig. 2). By contrast, Selective Memory Purge maintains a moderate Average Score. Pooling (triplicate runs) all Tanimoto similarities, one can see that Selective Memory Purge generates a broader distribution of high Tanimoto similarities in contrast to Diversity Filter (Fig. 2).

Table 1: Robustness experiments: stability of two augmentation rounds. 100 replicates of Aripiprazole Similarity was performed using 2 augmentation rounds and the epoch number to reach various average scores are presented. The values for Double Loop RL[1] are for 10 augmentations which the authors state to be most stable

Average Score	Mean Epochs	Double Loop RL Mean Epochs
0.5	65 ± 6	93 ± 9
0.8	99 ± 23	194 ± 31
0.9	122 ± 19	did not report

Pure Exploitation: Robustness of 2 Augmentation Rounds

Initial screening experiments identified two augmentation rounds to be optimal for training stability. We envisioned in **pure exploitation** scenarios where **Selective Memory Purge** is not used, mode collapse may be possible. The rationale being that the Agent is reinforced on the same replay buffer molecules. In the case where the entire replay buffer contains very similar or identical molecules, mode collapse may occur. **This is not an issue when using Selective Memory Purge as entries in the replay buffer would be removed, thus preventing the entire buffer containing the same molecules. We verified this statement by performing 100 replicates of Aripiprazole Similarity using Selective Memory Purge. All replicates rediscovered Aripiprazole, indicating no mode collapse at sub-optimal minima.**

In most practical applications of molecular generative models, Selective Memory Purge should be used to achieve both exploration and exploitation. However, for full transparency, we report the stability of our proposed method in a pure exploitation scenario. The following insights will be informative if prospective users only want to generate one optimal solution in their generative experiment or want to reproduce the Aripiprazole Similarity experiment. To preemptively prevent mode collapse, we introduce "mode collapse guard" that purges the replay buffer if 50 percent of the buffer contains the exact same reward. For statistical rigour, we perform 100 replicates of Aripiprazole Similarity and present the results in Table 1. We follow Bjerrum et al.[1] and present statistics on the epochs it takes to reach various average

scores (average Tanimoto similarity of the batch of sampled molecules to aripiprazole) of 0.5, 0.8, and 0.9. The results support the stability of our method even in pure exploitation scenarios. The "mode collapse guard" was activated 16 times across 100 replicates and in all cases except 4, prevents mode collapse. The exceptions failed to rediscover aripiprazole (mode collapse at a Tanimoto similarity of 0.88). In practical applications, the experiment can be monitored and restarted from a check-point state. Moreover, we comment on the number of epochs it takes to reach an average score of 0.8 and 0.9 which are, in both cases, much lower than Double Loop RL.[1] In particular, even at three standard deviations from the mean, the epochs it takes our method is faster than Double Loop RL. We further note that it is unclear if running their algorithm for 100 replicates would still be stable as it is not open-sourced. Overall, in pure exploitation scenarios, mode collapse can be observed but is not a problem if Selective Memory Purge is used. We highlight the practical usage of Augmented Memory with Selective Memory Purge to generate diverse molecules on the DRD2 case study in the main results. Our method is consistently stable.

We end this section by further discussing the choice to use a "mode collapse guard" and why it is not just to make our method look more robust. The insights in this section are for transparency on our method but also positions the work for future integration with curriculum learning (CL).[3,4] In curriculum learning, a complex objective function is decomposed into simpler sequential tasks to accelerate the learning process. It may be advantageous to enforce pure exploitation for intermediate tasks as the objective is to learn as quickly as possible this task before moving to the successive task. In this case, enforcing diversity could be counterproductive. While mode collapse is rare, as discussed above, its possibility decreases the robustness of a direct CL integration. Leveraging the insights from this section, a lenient Selective Memory Purge (allowing a larger number of identical scaffolds to be sampled before penalization) can be applied on top of CL to retain Augmented Memory's sample efficiency while also capitalizing on benefits of CL.

Buffer Size Experiments and Reinforcing with Only Experience Replay

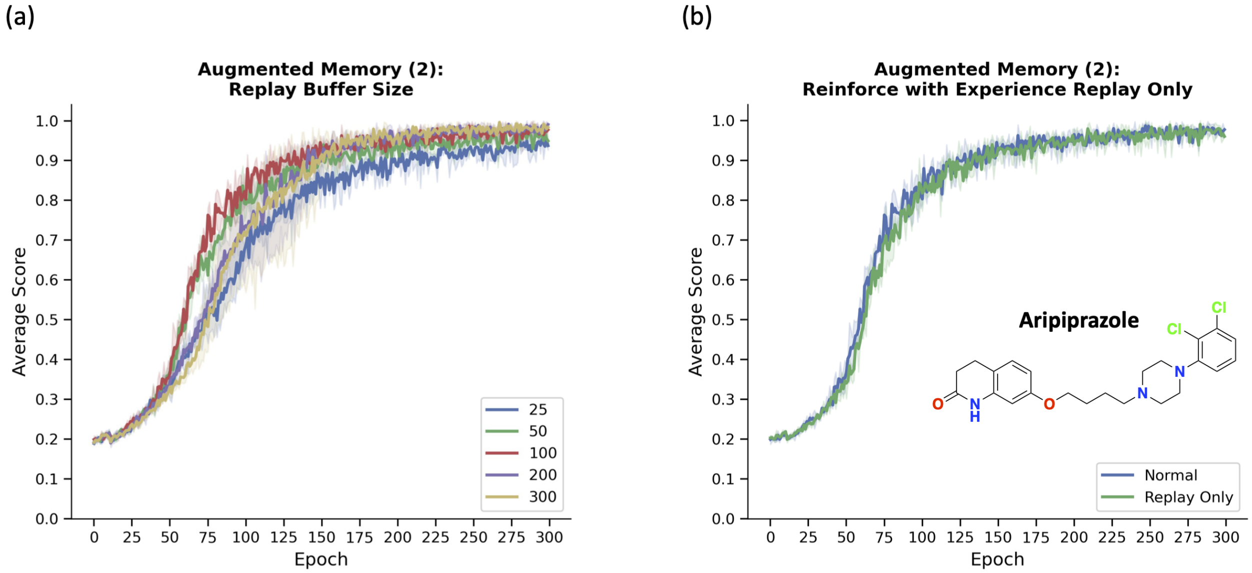

Figure 3: Investigating changes in the replay buffer size and reinforcing the Agent only with molecules stored in the replay buffer. The shaded region represents the minimum and maximum scores across triplicate runs.

As Augmented Memory revolves around exploiting experience replay, we investigate the efficacy of our method when using different buffer sizes (Figure 3). We again use the Aripiprazole Similarity task to assess the proposed changes. The results show that smaller (25) and larger (200 and 300) buffer sizes decreases the sample efficiency (Figure 3). We posit that the former is due to fewer high rewarding examples being used to reinforce the Agent. Conversely, larger buffer sizes would be less selective towards high rewarding molecules and reinforcing the Agent with relatively low rewarding molecules can be counterproductive. Interestingly, however, the differences are not drastic. Given that the differences in the buffer sizes result in minimal difference and that our hypothesis may be true for other objective functions, we decided to use a buffer size of 100 for main result experiments. Next, we were curious if reinforcing the Agent with only the molecules in the replay buffer would be possible. In these experiments, the sampled molecules in a given epoch were only used to reinforce

the Agent once and no augmented forms were used to further reinforce the Agent. Minimal difference is observed again (Figure 3). Since the performance is similar, we hypothesize that using augmented forms of the sampled molecules would act to mitigate mode collapse. This is in agreement with insights from Arús-Pous et al.[5] that posit SMILES augmentation acts as a regularizer. Therefore, all main result experiments were performed using augmented SMILES from the sampled batch and the buffer. We expand upon these observations in the ablation study in the next section.

Ablation Study: SMILES Augmentation is a Regularizer

Table 2: Stability without SMILES augmentation. 100 replicates of Aripiprazole Similarity was performed using 2 augmentation rounds (but without SMILES augmentation) and the epoch number to reach various average scores are presented. Failed runs did not reach the average score threshold. The epoch numbers for the runs with augmentation are shown in parenthesis for comparison.

Average Score	Mean Epochs	Failed Runs
0.5	$69(65) \pm 8(6)$	0
0.8	$119(99) \pm 41(23)$	3
0.9	$159(122) \pm 47(19)$	14

The results in the main text show that experience replay is vital for sample efficiency. In this section, the question we answer is: "can we just perform multiple rounds of Agent update with the entire replay buffer without SMILES augmentation?" If yes, then the benefits of Augmented Memory can be attributed to simply experience replay. The experimental design is as follows: using the Aripiprazole Similarity experiment, perform two rounds of Agent update using the entire buffer (size of 100) without SMILES augmentation. This mirrors the optimal parameters of Augmentation Memory of two augmentation rounds and a buffer size of 100. For statistical rigour, we perform 100 replicates and present the results in Table 2. Compared to Augmented Memory, the average epochs it takes to reach an average score of 0.5, 0.8, and 0.9 is higher (values with augmentation are shown in parentheses and is from

Table 1). Importantly, the standard deviation is also much higher, suggesting instability in the runs. This is further supported by some runs not reaching the 0.8 and 0.9 average score thresholds. While monitoring the sampling, we notice that the Agent repeatedly samples the same SMILES, indicating mode collapse. From a probabilistic perspective, the Agent negative log-likelihoods (NLLs) become focused on the replay buffer sequences, suggesting token-level memorization. These insights are supported by previous work from Arús-Pous et al.[5] which explored the effect of SMILES augmentation on the Prior's NLL on the training data. Specifically, they found that training a Prior without SMILES augmentation can cause token-level memorization, such that the NLL for the specific SMILES sequences in the training data are low. This decreases the generalizability of the trained Prior. Bjerrum et al.[1] in their Double Loop RL work also posit that reinforcing the Agent on augmented SMILES prevents sequence-wise mode collapse. Our results are in agreement and we show that SMILES augmentation is necessary to ensure the efficacy of Augmented Memory and is itself a regularizer.

Dopamine Type 2 Receptor (DRD2) Case Study: Exploiting AutoDock Vina

This section elaborates on the statement that the experimental design of Bjerrum et al.[1] in their Double Loop RL work is insufficient in preventing AutoDock Vina[6] exploitation. Specifically, the drug discovery case study to design potential dopamine type 2 receptor (DRD2) inhibitors was performed using the following objective function: molecular weight (MW) $<$ 500 Da, maximize QED, and minimize docking score. This is in contrast to the objective function proposed by Bjerrum et al.:[1] molecular MW $<$ 500 Da and minimize docking score. We perform a set of experiments comparing the sample efficiency of alternative algorithms including REINVENT,[7,8] Best Agent Reminder (BAR),[9] and Augmented Hill Climbing (AHC)[10] using this simplified objective function. Similar to the main result

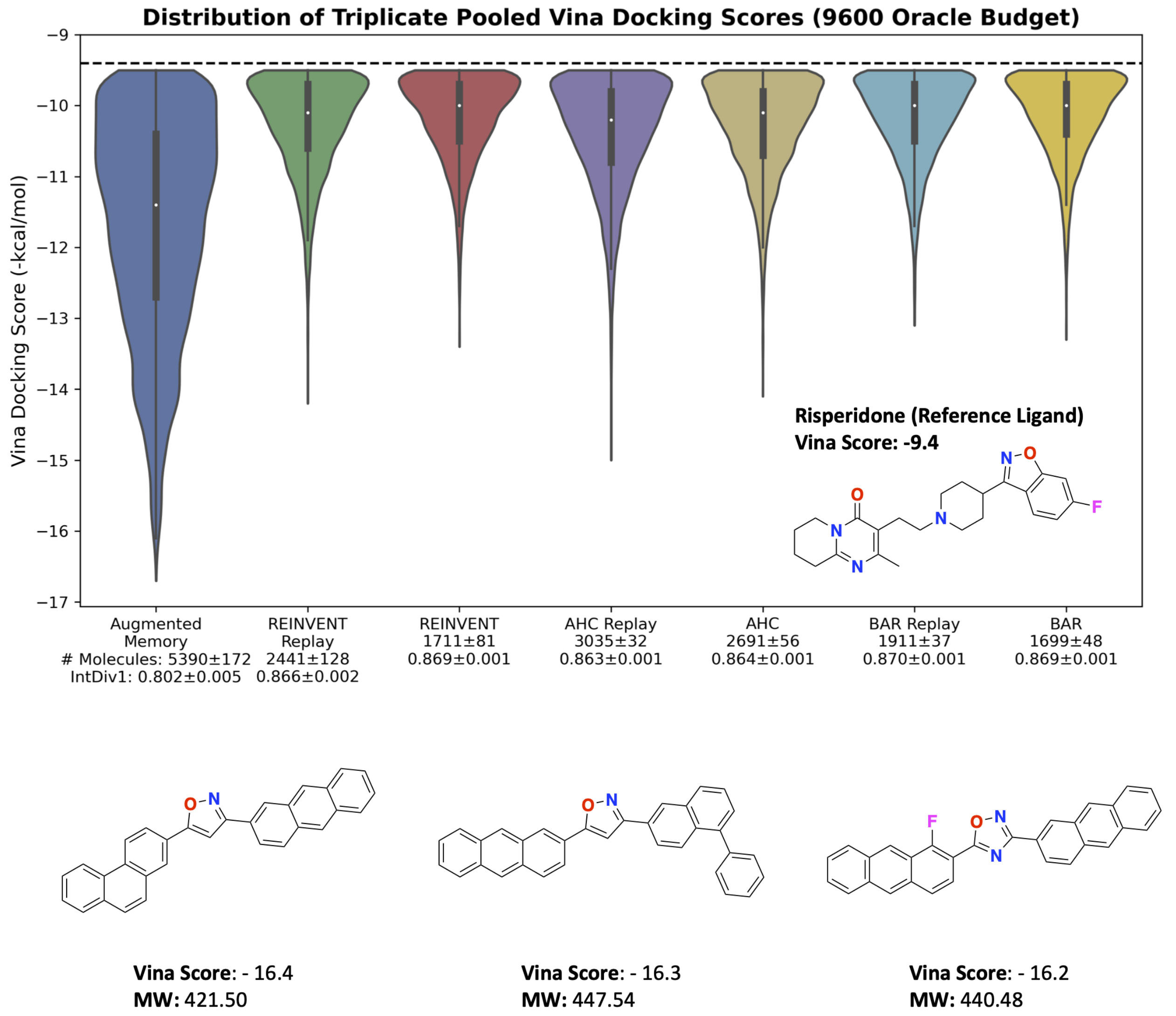

Figure 4: Dopamine type 2 receptor (DRD2) case study using the objective function: molecular weight $<$ 500 Da and minimize Vina docking score. Augmented Memory significantly outperforms other algorithms. The generated molecules, however, are not realistic and shows that Augmented Memory can exploit objective functions in a sample efficient manner.

experiments, we ran all experiments enforcing an oracle budget of 9,600 calls and show the distribution of Vina scores from triplicate pooled runs. The following filter was applied: MW $<$ 500 Da and Vina score $<$ -9.4 (the Vina score of the reference drug molecules, risperidone). It is evident that Augmented Memory significantly outperforms other algorithms, generating drastically better docking scores and more molecules passing the filter. Moreover, experience replay improves the performance of all base algorithms. However, we investigate the property profile of the generated molecules in the Augmented Memory experiments and show that the Agent exploits AutoDock Vina in rewarding lipophilic molecules, i.e., all top scoring molecules have extensive aromatic carbon rings (Figure 4). These molecules, while possessing excellent Vina scores, are not realistic. As we emphasize the usability of Augmented Memory on more realistic case studies to encourage practical applications, we show the set of experiments which also enforce QED[11] in the main results. QED ensures generated molecules are "drug-like". We end this section by emphasizing that the ability of Augmented Memory to exploit AutoDock Vina is not a weakness and rather, further proves its ability for sample efficient optimization.

Dopamine Type 2 Receptor (DRD2) Case Study: Increased Oracle Budget (19,200)

In this section, we run the DRD2 case study with the following MPO objective: molecular weight (MW) $<$ 500 Da, maximize QED,[11] and minimize Vina[6] docking score with an oracle budget of 19,200 which is two times the budget of the main text experiment. The purpose is to show that the other algorithms do not catch up to Augmented Memory's sample efficiency with an increased budget. Following the filter used in the main text experiments, Fig. 5 shows the number of molecules generated that have MW $<$ 500 Da, QED $>$ 0.4 (risperidone's QED is 0.66), and Vina score $<$ -9.4 kcal/mol (risperidone's docking score). Augmented Memory generates almost three times more molecules passing the filter compared with the other algorithms *with* experience replay.

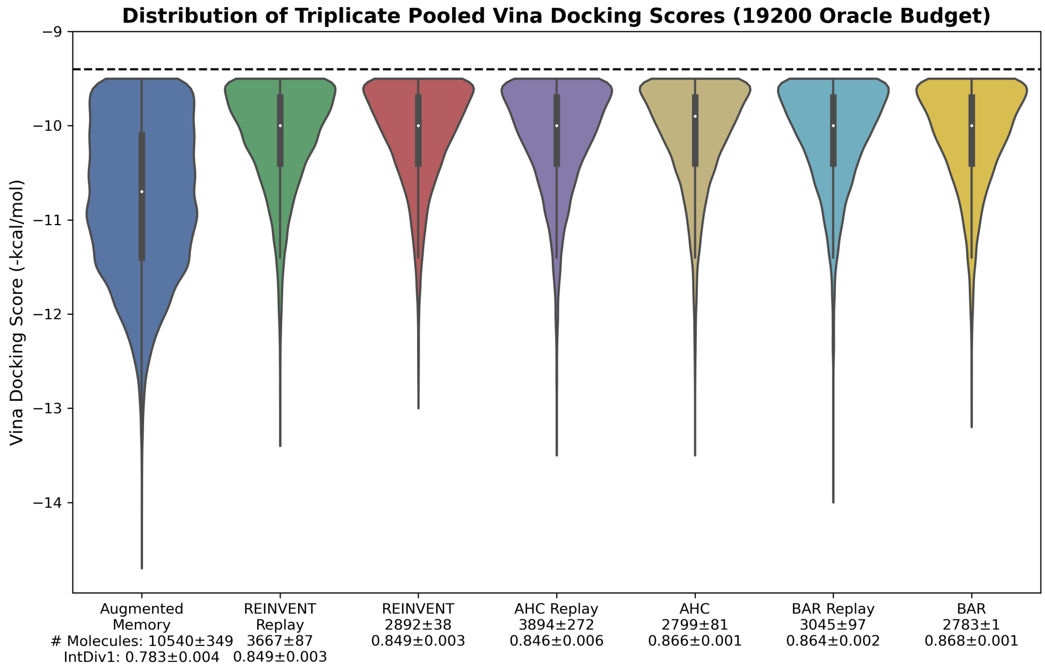

Figure 5: Dopamine type 2 receptor (DRD2) case study using the objective function: molecular weight $<$ 500 Da, maximize QED, and minimize Vina docking score. The oracle budget is increased to 19,200 calls. Augmented Memory significantly outperforms other algorithms and this benefit does not flatten out with the increased oracle budget.

Aripiprazole and DRD2 Prior and Hyperparameters

The *random.prior.new* pre-trained Prior was used from the REINVENT 2.0[8] repository which was trained on ChEMBL.[12] We note that for the Aripiprazole Similarity experiment, this enables direct comparison to Double Loop RL[1] as the authors also used the same Prior. The hyperparameters used for Experiment 1: Aripiprazole Similarity and Experiment 3: Dopamine Type 2 Receptor (DRD2) are presented in Table 3.

Table 3: Hyperparameters (default) used in Experiment 1: Aripiprazole Similarity and Experiment 3: Dopamine Type 2 Receptor (DRD2).

Algorithm	Sigma (σ)	Batch Size	Learning Rate	k	Alpha (α)
Augmented Memory	128	64	0.0001	N/A	N/A
REINVENT[7,8]	128	64	0.0001	N/A	N/A
Augmented Hill-Climbing (AHC)[10]	60	64	0.0001	0.5	N/A
Best Agent Reminder (BAR)[9]	1	64	0.0001	N/A	0.5

Practical Molecular Optimization (PMO) Hyperparameters

Table 4: Hyperparameters used in Experiment 2: Practical Molecular Optimization (PMO)[13] Benchmark.

Algorithm	Sigma (σ)	Batch Size	Learning Rate	k	Alpha (α)
Augmented Memory	500	64	0.0005	N/A	N/A
REINVENT[7,8]	500	64	0.0005	N/A	N/A
Augmented Hill-Climbing (AHC)[10]	120	256	0.0005	0.25	N/A
Best Agent Reminder (BAR)[9]	1000	64	0.0005	N/A	0.25

The hyperparameters used for the Practical Molecular Optimization (PMO)[13] benchmark is presented in Table 4. The hyperparameters provided in the PMO repository for REINVENT[7,8] and AHC[10] were used. The hyperparameters for BAR[9] were tuned according to the protocol provided in the PMO[13] paper and the results are presented in Table 5. We note

Table 5: Best Agent Reminder (BAR)[9] hyperparameter tuning for Experiment 2: Practical Molecular Optimization (PMO).[13] The AUC top-10 was used to assess performance and was based on the protocol proposed in the PMO benchmark: average AUC top-10 across 3 independent runs of zaleplon_mpo and perindopril_mpo.

Sigma (σ)	Alpha (α)	Top-10 AUC
250	0.25	0.610
250	0.50	0.677
500	0.25	0.708
500	0.50	0.728
750	0.25	0.739
750	0.50	0.732
**1000**	**0.25**	**0.762**
1000	0.50	0.759

that the default σ hyperparameter is 1 as stated in the BAR repository. However, we found that the resulting AUC Top-10 was much lower than all σ values in 5. Thus, we performed hyperparameter tuning using much larger σ values according to the values REINVENT was tuned with.

Practical Molecular Optimization (PMO) Augmented Memory and BAR Prior

Table 6: LSTM model hyperparameters for Augmented Memory and BAR

Cell Type	LSTM
Number of Layers	3
Embedding Layer Size	256
Dropout	0
Training Batch Size	128
SMILES Training Randomization	True

Augmented Memory required training a Prior and follows the protocol from REINVENT[8] and using the provided ZINC[14] dataset in the PMO[13] repository. Table 6 shows the hyperparameters of the LSTM[15] network. We note all hyperparameters were kept default and the

model was trained for 10 epochs as SMILES validity reached 95% and the total wall time was 11 minutes 57 seconds. BAR[9] experiments were run with this same pre-trained Prior.

DRD2 Experiment Wall Times

Table 7: Experiment 3: Dopamine Type 2 Receptor (DRD2) Case Study Wall Times.

Algorithm	Wall Time (9,600 Budget)	Wall Time (19,200 Budget)
Augmented Memory	21h 25m ± 2h 20m	31h 53m ± 0h 8m
REINVENT	27h 7m ± 2h 21m	43h 9m ± 1h 16m
Augmented Hill-Climbing (AHC)	30h 24m ± 4h 48m	45h 48m ± 2h 7m
Best Agent Reminder (BAR)	35h 18ms ± 2h 12m	54h 38m ± 4h 51m

The wall times for Experiment 3: Dopamine Type 2 Receptor (DRD2) are presented in Table 7. We note that we performed a total of 6 replicates for each algorithm: 3 in the main result experiments and 3 in the exploiting AutoDock Vina experiments (Figure 4). For REINVENT,[7,8] AHC,[10] and BAR,[9] we pool the experiments using experience replay. For example, REINVENT values are reported based on 12 total runs: 3 for main result experiments, 3 for main results experiments with experience replay, 3 for exploiting AutoDock Vina experiments, and 3 for exploiting AutoDock Vina experiments with experience replay. The exception is the wall times for the 19,200 oracle budget experiments. Since exploitative AutoDock Vina experiments were not run with this budget, they are not included in the averaging. The bottleneck in all experiments is AutoDock Vina[6] and the wall time is highly variable, depending on the molecules sampled by the Agent. Finally, we note that all experiments were run with a batch size of 64 for 150 epochs. The exception is BAR which was run for 75 epochs as each epoch samples 2 batches of molecules: one from the current Agent and one from the best Agent. Finally, we comment on the variable wall times of each algorithm despite having a fixed oracle budget. There are two sources of stochasticity. Firstly, the experiments were performed on a shared cluster and compute speed is variable depending

on usage. Secondly, docking is itself stochastic and generally requires more search time for larger molecules. Augmented Memory jointly optimizes for Vina, QED,[11] and molecular weight, which generally enforces smaller molecules and could be a reason for the faster average compute time.

AutoDock Vina DRD2 Receptor Preparation and Docking

The receptor grid for AutoDock Vina[6] docking against DRD2 (PDB ID: 6CM4[16]) was performed using DockStream.[17] The PDB file for 6CM4 was first downloaded from the Protein Data Bank. One monomer unit was extracted and refined using PDBFixer[18] through the DockStream wrapper. The prepared grid was centered at (x, y, z) = (9.93, 5.85, -9.58) with a search box of 15Å x 15Å x 15Å. Docking for all experiments were performed with DockStream using the following protocol: embed sampled SMILES with RDKit Universal Force Field (UFF)[19] with 600 maximum convergence iterations and execute AutoDock Vina docking parallelized over 36 CPU cores (Intel(R) Xeon(R) Platinum 8360Y processors).

Optoelectronics Experiment Wall Times

Table 8: Experiment 4: Optoelectronics: Designing Out-of-Distribution Wall Times.

Algorithm	Wall Time (5,000 Budget)
Augmented Memory	2h 38m ± 0h 3m
Augmented Hill-Climbing (AHC) with Experience Replay	1h 57m ± 0h 5m

The wall times for Experiment 4: Optoelectronics are presented in Table 8. The mean and standard deviation are shown across triplicate runs.

Proof of Loss Function and Policy Gradient Equivalency

In this section, we show that the loss function used to tune the Agent is equivalent to optimizing the expected reward of the policy following the REINFORCE[20] algorithm. Molecules are represented as a sequence of tokens given by the Simplified Molecular Input Line Entry System (SMILES)[21] format and generated in an autoregressive manner. The generative process is Markovian (Equation 1):

$$P(x) = \prod_{t=1}^{T} P(a_t \mid s_t) \tag{1}$$

Equation 1 states that the probability of generating a given SMILES, x, is equal to the product of the probabilities of generating a token at time-step t, given the sequence so far at time-step $t-1$. The model is pre-trained on a dataset of molecules (ChEMBL[12] for the DRD2 experiment, ZINC[14] for the benchmarking experiment, and OE62[22] for the optoelectronics experiment) to yield the Prior which is parameterized by the weights θ. The Agent is initialized identical to the Prior but is fine-tuned during the reinforcement learning (RL) process. The Augmented Likelihood is defined as a linear combination between the Prior and a reward term (Equation 2):

$$\log \pi_{\theta_{\text{Augmented}}} = \log \pi_{\theta_{\text{Prior}}} + \sigma S(x) \tag{2}$$

S is the reward function assessing the desirability of a sampled molecule and σ is a hyperparameter that scales the reward. A higher σ places a greater contribution on the reward function and less on the Prior. The Prior is used to ensure generated SMILES are syntactically correct and has been empirically shown to enforce reasonable chemistry. The loss function is defined as the squared difference between the Augmented Likelihood and the Agent Likelihood for a given batch, B, of sampled SMILES constructed following the actions, $a \in A^*$ (Equation 3):

$$L(\theta) = \frac{1}{|B|}\left[\sum_{a\in A^*}(\log \pi_{\theta_{\text{Augmented}}} - \log \pi_{\theta_{\text{Agent}}})\right]^2 \tag{3}$$

Taking the derivative with respect to θ (Equation 4):

$$\nabla_\theta L(\theta) = -2\frac{1}{|B|}\left[\sum_{a\in A^*}\log \pi_{\theta_{\text{Augmented}}} - \log \pi_{\theta_{\text{Agent}}}\right]\sum_{a\in A^*}\nabla_\theta \log \pi_{\theta_{\text{Agent}}} \tag{4}$$

Minimizing $J(\theta)$ tunes the Agent to generate molecules satisfying the reward function.

Following Fialková et al.,[23] we now show that minimizing $J(\theta)$ is equivalent to optimizing the expected reward of the policy. The generative process is cast as an on-policy RL problem by defining the state space, S_t, and the action space, $A_t(s_t)$. S_t denotes every intermediate sequence of tokens leading up to the fully constructed SMILES and $A_t(s_t)$ are the token sampling probabilities at every intermediate state. $A_t(s_t)$ is controlled by the policy, π_θ, which is parameterized by the weights, θ, of the neural network. Given a reward function, R, the objective is to maximize the expected reward when taking actions defined by the policy (Equation 5):

$$J(\theta) = \mathbb{E}_{a_t\sim\pi_\theta}\left[\sum_{t=0}^{T} R(a_t, s_t)\right] \tag{5}$$

Rewriting the expectation (Equation 6):

$$J(\theta) = \sum_{t=0}^{T}\sum_{a\in A_t} R(a_t, s_t)\pi_\theta(a_t|s_t) \tag{6}$$

The expectation can be rewritten as a double summation over all time-steps and actions taken at each time-step, following the policy, π_θ. Next, the derivative of the expression is taken (Equation 7):

$$\nabla_\theta J(\theta) = \sum_{t=0}^{T}\sum_{a\in A_t} R(a_t, s_t)\nabla_\theta\pi_\theta(a_t|s_t) \tag{7}$$

Applying the log-derivative trick (Equation 8):

$$\nabla_\theta J(\theta) = \sum_{t=0}^{T} \sum_{a \in A_t} R(a_t, s_t) \pi_\theta(a_t|s_t) \nabla_\theta \log \pi_\theta(a_t|s_t) \tag{8}$$

Using the definition of expectation for discrete variables, i.e., the policy actions which can only sample the vocabulary tokens (Equation 9):

$$\nabla_\theta J(\theta) = \mathbb{E}_{a_t \sim \pi_\theta} \left[\sum_{t=0}^{T} R(a_t, s_t) \nabla_\theta \log \pi_\theta(a_t|s_t) \right] \tag{9}$$

As computing the expectation is intractable, it is instead approximated by sampling a batch, B, of trajectories, i.e., SMILES strings. The process of SMILES generation is further defined as an episodic task where reward is only given at the terminal state. In particular, the *desirability* of a SMILES sequence only applies once the full SMILES string has been sampled and it maps to a valid molecule. Thus, all intermediate rewards are 0. Defining the set of actions taken in a batch, A^* as the specific token sequences generated at a given epoch yields Equation 10:

$$\nabla_\theta J(\theta) = \frac{1}{|B|} \left[\sum_{t=0}^{T} \sum_{a \in A^*} R(a_t, s_t) \nabla_\theta \log \pi_\theta(a_t|s_t) \right] \tag{10}$$

Finally, the reward, R is defined according to Fialková et al.[23] (Equation 11):

$$R(a_t, s_t) = \log \pi_{\theta_{\text{Augmented}}} - \log \pi_{\theta_{\text{Agent}}} \tag{11}$$

Substituting Equation 11 into Equation 10 yields the desired equivalency to the loss function (Equation 4) up to a constant factor.

Augmented Memory Algorithm

The pseudo-code for Augmented Memory is presented here.

Algorithm 1: Augmented Memory

Input: Prior π_{Prior}, Epochs N, Augmentation Rounds A, Scoring Function S, Sigma σ

Output: Fine-tuned Agent Policy $\pi_{\theta_{\text{Agent}}}$, Generated Molecules G

Initialization:

Generative Agent $\pi_{\theta_{\text{Agent}}} = \pi_{\text{Prior}}$;

Diversity Filter DF;

Replay Buffer $B = \{\}$;

Replay Buffer Size K;

for $i \leftarrow 1$ **to** N **do**

  Sample batch of SMILES $X = \{x_1, \ldots, x_b\}$ with $x_i \sim \pi_{\theta_{\text{Agent}}}$;

  Compute reward using the scoring function $S(X)$;

  Modify reward based on the diversity filter $DF(S(X))$;

  Update replay buffer $B_i = TopK(X_i \cup B_{i-1})$;

  (Optionally) purge replay buffer;

  Compute Augmented Likelihood $\log \pi_{\theta_{\text{Augmented}}} = \log \pi_{\text{Prior}}(X) + \sigma S(X)$;

  Compute loss $J(\theta) = (\log \pi_{\text{Augmented}} - \log \pi_{\theta_{\text{Agent}}}(X))^2$;

  Update the Agent's policy $\pi_{\theta_{\text{Agent}}}$;

  for $j \leftarrow 1$ **to** A **do**

    Augment sampled SMILES $X_{\text{Augmented}}$;

    Compute Augmented Likelihood of augmented SMILES (reward is unchanged) $\log \pi_{\text{Augmented}} = \log \pi_{\text{Prior}}(X_{\text{Augmented}}) + \sigma S(X)$;

    Compute loss $J(\theta)_{\text{Augmented}} = (\log \pi_{\text{Augmented}} - \log \pi_{\theta_{\text{Agent}}}(X_{\text{Augmented}}))^2$;

    Augment entire replay buffer $B_{\text{Augmented}}$;

    Compute Augmented Likelihood on the augmented buffer (reward is the buffer stored rewards) $\log \pi_{\text{Buffer Augmented}} = \log \pi_{\text{Prior}}(B_{\text{Augmented}}) + \sigma S(B)$;

    Compute augmented buffer loss $J(\theta)_{\text{Buffer Augmented}} = (\log \pi_{\text{Buffer Augmented}} - \log \pi_{\theta_{\text{Agent}}}(B_{\text{Augmented}}))^2$;

    Concatenate the augmented sampled SMILES loss and the augmented buffer loss $J(\theta)_{\text{Augmented Memory}} = J(\theta)_{\text{Augmented}} + J(\theta)_{\text{Buffer Augmented}}$;

    Update the Agent's policy $\pi_{\theta_{\text{Agent}}}$;

References

(1) Bjerrum, E. J.; Margreitter, C.; Blaschke, T.; Kolarova, S.; de Castro, R. L.-R. Faster and more diverse de novo molecular optimization with double-loop reinforcement learning using augmented SMILES. *Journal of Computer-Aided Molecular Design* **2023**, 1–22.

(2) Bemis, G. W.; Murcko, M. A. The properties of known drugs. 1. Molecular frameworks. *Journal of medicinal chemistry* **1996**, *39*, 2887–2893.

(3) Bengio, Y.; Louradour, J.; Collobert, R.; Weston, J. Curriculum learning. Proceedings of the 26th annual international conference on machine learning. 2009; pp 41–48.

(4) Guo, J.; Fialková, V.; Arango, J. D.; Margreitter, C.; Janet, J. P.; Papadopoulos, K.; Engkvist, O.; Patronov, A. Improving de novo molecular design with curriculum learning. *Nat Mach Intell* **2022**, *4*, 555–563, Number: 6 Publisher: Nature Publishing Group.

(5) Arús-Pous, J.; Johansson, S. V.; Prykhodko, O.; Bjerrum, E. J.; Tyrchan, C.; Reymond, J.-L.; Chen, H.; Engkvist, O. Randomized SMILES strings improve the quality of molecular generative models. *Journal of Cheminformatics* **2019**, *11*, 71.

(6) Trott, O.; Olson, A. J. AutoDock Vina: improving the speed and accuracy of docking with a new scoring function, efficient optimization, and multithreading. *Journal of computational chemistry* **2010**, *31*, 455–461.

(7) Olivecrona, M.; Blaschke, T.; Engkvist, O.; Chen, H. Molecular de-novo design through deep reinforcement learning. *Journal of Cheminformatics* **2017**, *9*, 48.

(8) Blaschke, T.; Arús-Pous, J.; Chen, H.; Margreitter, C.; Tyrchan, C.; Engkvist, O.; Papadopoulos, K.; Patronov, A. REINVENT 2.0: An AI Tool for De Novo Drug Design. *J. Chem. Inf. Model.* **2020**, *60*, 5918–5922, Publisher: American Chemical Society.

(9) Atance, S. R.; Diez, J. V.; Engkvist, O.; Olsson, S.; Mercado, R. De novo drug design

using reinforcement learning with graph-based deep generative models. *Journal of Chemical Information and Modeling* **2022**, *62*, 4863–4872.

(10) Thomas, M.; O'Boyle, N. M.; Bender, A.; de Graaf, C. Augmented Hill-Climb increases reinforcement learning efficiency for language-based de novo molecule generation. *Journal of Cheminformatics* **2022**, *14*, 68.

(11) Bickerton, G. R.; Paolini, G. V.; Besnard, J.; Muresan, S.; Hopkins, A. L. Quantifying the chemical beauty of drugs. *Nature Chem* **2012**, *4*, 90–98, Number: 2 Publisher: Nature Publishing Group.

(12) Gaulton, A.; Bellis, L. J.; Bento, A. P.; Chambers, J.; Davies, M.; Hersey, A.; Light, Y.; McGlinchey, S.; Michalovich, D.; Al-Lazikani, B.; Overington, J. P. ChEMBL: a large-scale bioactivity database for drug discovery. *Nucleic Acids Res* **2012**, *40*, D1100–D1107.

(13) Gao, W.; Fu, T.; Sun, J.; Coley, C. Sample efficiency matters: a benchmark for practical molecular optimization. *Advances in Neural Information Processing Systems Datasets and Benchmarks Track* **2022**, *35*, 21342–21357.

(14) Irwin, J. J.; Tang, K. G.; Young, J.; Dandarchuluun, C.; Wong, B. R.; Khurelbaatar, M.; Moroz, Y. S.; Mayfield, J.; Sayle, R. A. ZINC20—A Free Ultralarge-Scale Chemical Database for Ligand Discovery. *J. Chem. Inf. Model.* **2020**, *60*, 6065–6073, Publisher: American Chemical Society.

(15) Hochreiter, S.; Schmidhuber, J. Long short-term memory. *Neural computation* **1997**, *9*, 1735–1780.

(16) Wang, S.; Che, T.; Levit, A.; Shoichet, B. K.; Wacker, D.; Roth, B. L. Structure of the D2 dopamine receptor bound to the atypical antipsychotic drug risperidone. *Nature* **2018**, *555*, 269–273.

(17) Guo, J.; Janet, J. P.; Bauer, M. R.; Nittinger, E.; Giblin, K. A.; Papadopoulos, K.; Voronov, A.; Patronov, A.; Engkvist, O.; Margreitter, C. DockStream: a docking wrapper to enhance de novo molecular design. *Journal of Cheminformatics* **2021**, *13*, 89.

(18) Eastman, P.; Swails, J.; Chodera, J. D.; McGibbon, R. T.; Zhao, Y.; Beauchamp, K. A.; Wang, L.-P.; Simmonett, A. C.; Harrigan, M. P.; Stern, C. D.; others OpenMM 7: Rapid development of high performance algorithms for molecular dynamics. *PLoS computational biology* **2017**, *13*, e1005659.

(19) Rappé, A. K.; Casewit, C. J.; Colwell, K.; Goddard III, W. A.; Skiff, W. M. UFF, a full periodic table force field for molecular mechanics and molecular dynamics simulations. *Journal of the American chemical society* **1992**, *114*, 10024–10035.

(20) Williams, R. J. Simple statistical gradient-following algorithms for connectionist reinforcement learning. *Mach Learn* **1992**, *8*, 229–256.

(21) Weininger, D. SMILES, a chemical language and information system. 1. Introduction to methodology and encoding rules. *J. Chem. Inf. Comput. Sci.* **1988**, *28*, 31–36, Publisher: American Chemical Society.

(22) Stuke, A.; Kunkel, C.; Golze, D.; Todorović, M.; Margraf, J. T.; Reuter, K.; Rinke, P.; Oberhofer, H. Atomic structures and orbital energies of 61,489 crystal-forming organic molecules. *Scientific data* **2020**, *7*, 58.

(23) Fialková, V.; Zhao, J.; Papadopoulos, K.; Engkvist, O.; Bjerrum, E. J.; Kogej, T.; Patronov, A. LibINVENT: Reaction-based Generative Scaffold Decoration for in Silico Library Design. *J. Chem. Inf. Model.* **2022**, *62*, 2046–2063, Publisher: American Chemical Society.
